# Super hotspots and super coldspots in the repair of UV-induced DNA damage in the human genome

**DOI:** 10.1016/j.jbc.2021.100581

**Published:** 2021-03-23

**Authors:** Yuchao Jiang, Wentao Li, Laura A. Lindsey-Boltz, Yuchen Yang, Yun Li, Aziz Sancar

**Affiliations:** 1 Department of Biostatistics, Gillings School of Global Public Health, University of North Carolina, Chapel Hill, North Carolina, USA; 2 Department of Genetics, School of Medicine, University of North Carolina, Chapel Hill, North Carolina, USA; 3 Lineberger Comprehensive Cancer Center, University of North Carolina, Chapel Hill, North Carolina, USA; 4 Department of Biochemistry and Biophysics, School of Medicine, University of North Carolina, Chapel Hill, North Carolina, USA; 5 Department of Computer Science, College of Arts and Sciences, University of North Carolina, Chapel Hill, North Carolina, USA

**Keywords:** nucleotide excision repair, excision repair-sequencing, cyclobutane pyrimidine dimer, (6-4) pyrimidine–pyrimidone photoproduct, (6-4)PPs, (6-4) pyrimidine–pyrimidone photoproducts, CPD, cyclobutane pyrimidine dimer, FIREs, frequently interacting regions, GEO, Gene Expression Omnibus, Hi-C, high-throughput chromosome conformation capture, NGS, next-generation sequencing, NHF1, normal human skin fibroblast 1, NTS, nontranscribed strand, TADs, topologically associating domains, TCR, transcription-coupled repair, TFIIH, Transcription factor II H, TS, transcribed strand, XR-seq, eXcision Repair-sequencing

## Abstract

The formation of UV-induced DNA damage and its repair are influenced by many factors that modulate lesion formation and the accessibility of repair machinery. However, it remains unknown which genomic sites are prioritized for immediate repair after UV damage induction, and whether these prioritized sites overlap with hotspots of UV damage. We identified the super hotspots subject to the earliest repair for (6-4) pyrimidine–pyrimidone photoproduct by using the eXcision Repair-sequencing (XR-seq) method. We further identified super coldspots for (6-4) pyrimidine–pyrimidone photoproduct repair and super hotspots for cyclobutane pyrimidine dimer repair by analyzing available XR-seq time-course data. By integrating datasets of XR-seq, Damage-seq, adductSeq, and cyclobutane pyrimidine dimer-seq, we show that neither repair super hotspots nor repair super coldspots overlap hotspots of UV damage. Furthermore, we demonstrate that repair super hotspots are significantly enriched in frequently interacting regions and superenhancers. Finally, we report our discovery of an enrichment of cytosine in repair super hotspots and super coldspots. These findings suggest that local DNA features together with large-scale chromatin features contribute to the orders of magnitude variability in the rates of UV damage repair.

Nucleotide excision repair is a versatile repair pathway that removes a variety of bulky and helix-distorting lesions caused by DNA-damaging agents, such as UV, cisplatin, and benzo(a)pyrene (1, 2). It has two subpathways: global repair, which repairs DNA lesions throughout the whole genome, and transcription-coupled repair (TCR), which preferentially removes DNA lesions from the transcribed strand (TS) of transcriptionally active genes (3, 4). The two subpathways differ only in the damage recognition step and share the steps of dual incision bracketing the lesions, release of the excision products, repair synthesis, and ligation (5, 6).

UV-induced DNA damage, if not removed efficiently, will lead to mutations and possibly carcinogenesis in humans. UV in sunlight is a known mutagen and causative agent of skin cancer (7, 8), inducing DNA lesions, such as cyclobutane pyrimidine dimers (CPDs) and (6-4) pyrimidine–pyrimidone photoproducts [(6-4)PPs]. To better understand the molecular mechanisms of UV-induced mutagenesis and carcinogenesis, it is critical to identify the exact locations of DNA lesions and their repair efficiencies with single-nucleotide resolution on a genome-wide scale. With the advent of next-generation sequencing (NGS) technology, a number of NGS-based methods have been devised over the last 5 years to detect UV-induced DNA damage formation and repair across the whole genome (9), including Excision-seq (10), eXcision Repair-sequencing (XR-seq) (11), CPD-seq (12), translesion XR-seq (13), high-sensitivity Damage-seq (14), and adductSeq (15). Specifically, Damage-seq uses damage-specific immunoprecipitation and a high-fidelity DNA polymerase (which stops before the DNA damage during primer extension) to determine the exact positions of DNA damage (16); XR-seq directly measures the ongoing repair at a specific time point by isolating the excision products released during the repair for NGS (11, 17), and it has been successfully applied to generate genome-wide repair maps of UV damage with single-nucleotide resolution in humans (11), *Escherichia coli* (18), *Saccharomyces cerevisiae* (19), *Arabidopsis thaliana* (20), mice (21), *Drosophila melanogaster* (22), *Mycobacteria* (23), and *Microcebus murinus* (24).

Formation and repair of UV damage are influenced by multiple factors, including transcription (11), transcription factor binding (14, 25, 26, 27, 28), post-transcriptional modification of histones (29), nucleosome positioning (12), chromatin structure (29, 30), and 3D genome architecture (31). From the perspective of 3D genome organization, UV susceptibility generally is inversely correlated with chromatin accessibility (31). At the nucleosome level, however, CPDs favor the outward-facing rotation setting in a nucleosome, and (6-4)PPs tend to form in nucleosome linker regions (12, 30). This is because the outward-facing rotation setting in a nucleosome has conformational flexibility to accommodate a CPD, and such flexibility does not alter the DNA structure dramatically. In contrast, (6-4)PP formation requires greater DNA structure distortion; the nucleosome structure has no conformational flexibility for a (6-4)PP, except in linker regions. Depending on the nature of the individual transcription factor and the DNA-damaging agent, binding of a transcription factor to DNA may stimulate, inhibit, or have no effect on DNA damage formation (14, 25).

For repair of UV damage, the accessibility of repair machinery plays an important role. Repair occurs earlier in open chromatin regions than in repressed regions (29), and late repair regions, such as heterochromatic regions and some transcription factor binding sites, are associated with higher mutation rates (27, 29, 32, 33). We compared UV damage maps with repair maps and found that UV-induced DNA damage, measured with low depth of coverage, is uniformly distributed at a large-scale level and that the overall repair in the human genome is heterogeneous (14, 29). A recent study reported CPD hyper hotspots located near genes in human melanocytes and fibroblasts and suggested that these hyper hotspots may drive direct physiological changes rather than cause rare mutations (15).

Despite recent progress in DNA damage formation and repair research, it is still unknown which genomic sites are prioritized for repair immediately after UV irradiation and whether those prioritized sites overlap hotspots of DNA damage. Furthermore, determining which genomic sites are subject to nucleotide excision repair at very late stages of damage removal will offer additional insight into the question.

In this study, we sought to identify these genomic sites. We performed (6-4)PP XR-seq at 1 min and 2 min after UV treatment and integrated previously published data, which include (6-4)PP XR-seq ranging from 5 min to 4 h (11, 29) and CPD XR-seq as early as 12 min (22) following UV irradiation. Using these methods, we identified repair super hotspots and super coldspots for (6-4)PPs and repair super hotspots for CPDs. By comparing these repair super hotspots and super coldspots with other high-throughput sequencing datasets that measure UV damage formation, we showed that neither repair super hotspots nor super coldspots overlap hotspots of UV damage. Moreover, we demonstrated that repair super hotspots are significantly enriched in both frequently interacting regions (FIREs) and superenhancers. We also found an enrichment of cytosine in both repair super hotspots and super coldspots. Our findings suggest that both local chromatin structures (*e.g.*, transcription factor binding and previously assembled repair machinery members in the proximity of super hotspots) and large-scale chromatin features make it feasible for DNA damage to be rapidly removed in repair super hotspots. This effective integrity maintenance at repair super hotspots may confer a selective advantage.

## Results

### Profiling excision repair kinetics and UV damage formation

To identify which genomic sites are prioritized for nucleotide excision repair immediately after UV irradiation and which sites are subject to repair only at the latest stage of DNA damage removal, we designed an experimental and analytical framework to systematically investigate excision repair kinetics and UV damage formation over a time course. Removal of (6-4)PP occurs mainly through global repair and is completed within 4 h after UV irradiation (29, 34, 35). However, the removal of CPD requires both global repair and TCR, and the entire process takes days to complete (11, 29, 35). We have shown that global repair dominates CPD removal in the first 12 min after UV irradiation in normal human skin fibroblast 1 (NHF1) cells, and then at later time points, TCR also facilitates CPD removal (22). To avoid the confounding effects of transcription levels and TCR, we chose to focus on global repair of CPD and thus identified prioritized genomic sites for CPD repair in the first 12 min after UV irradiation.

**Figure 1:**
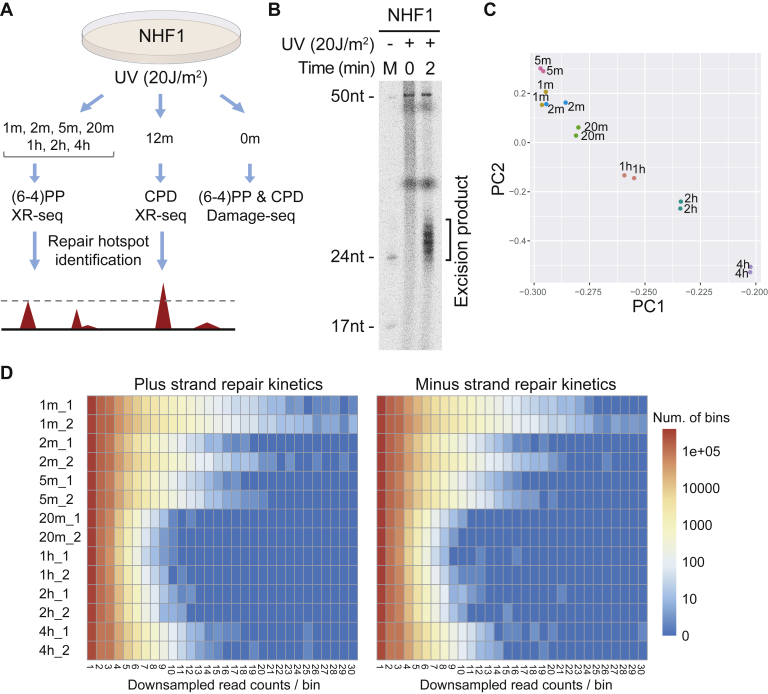
**Experimental design and identification of repair super hotspots and super coldspots.***A*, experimental design to measure UV damage formation and excision repair kinetics across different time points. *B*, detection of excision products at 0 and 2 min time points *in vivo*. Following UV irradiation, the excised oligonucleotides were purified by TFIIH immunoprecipitation, radiolabeled, and resolved in a 10% sequencing gel. DNA excision products containing (6-4)PP can be detected as early as 2 min upon damage induction. *C*, principal component (PC) analysis of genome-wide excision repair as measured by XR-seq shows repair kinetics across different time points. Between 1 and 5 min, excised oligonucleotides were not degraded, and XR-seq therefore measured cumulative repair. *D*, distributions of read counts per genomic bin across all (6-4)PP XR-seq samples. Each row is a sample, and each column is a specific total number of reads per genomic bin. The color in the heat map corresponds to the log counts of the number of bins with specific read depths. Early repair hotspots exist in samples collected at early time points. (6-4)PP, (6-4) pyrimidine–pyrimidone photoproduct; CPD, cyclobutane pyrimidine dimer; NHF1, normal human skin fibroblast 1; XR-seq, eXcision Repair-sequencing.

Figure 1*A* shows an outline of the experimental design we used to measure excision repair kinetics and UV damage formation. Specifically, we performed (6-4)PP XR-seq at 1 and 2 min after 20 J/m^2^ UV treatment in NHF1 and adopted previous NHF1 XR-seq data for (6-4)PP repair at 5 min, 20 min, 1 h, 2 h, and 4 h (11, 29) and CPD repair at 12 min (22). Refer to Table S1 for detailed XR-seq sample information. Damage-seq for both (6-4)PPs and CPDs at 0 min in NHF1 cells (14) was also included to determine the distribution of initial UV damage formation. Because release and degradation of excision products occur simultaneously and XR-seq does not measure the absolute number of excision products over time intervals (11, 34), it is necessary to perform XR-seq as early as possible to identify genomic sites that are subject to excision repair immediately after UV treatment. To determine the earliest time point and the optimal number of cells suitable for (6-4)PP XR-seq, we first performed *in vivo* excision assay at 0 and 2 min in NHF1 cells (Fig. 1*B*). As shown in Figure 1*B*, the primary excision products, ranging from 23 to 30 nt, can be seen at 2 min, but there are no degradation products at this time point and no signal at 0 min after UV treatment.

Based on this excision assay, we performed the (6-4)PP XR-seq at 1 and 2 min to identify genomic sites subject to immediate repair after UV treatment. Analyses of the two biological replicates for (6-4)PP XR-seq show high reproducibility (Fig. S1). As expected, length distribution and nucleotide frequency for reads from (6-4)PP XR-seq (1 and 2 min) and CPD XR-seq (12 min) are in agreement with previously reported data (Fig. S2) (11). Moreover, the TS/(TS + nontranscribed strand [NTS]) repair ratios in (6-4)PP XR-seq (1 min and 4 h) are on par with that in CPD XR-seq (12 min), indicating that the vast majority of DNA damage is removed by global repair by these time benchmarks (Fig. S3) (14, 22).

Using genome-wide repair data from XR-seq, we performed principal component (PC) analysis (36) on the top 2000 highly variable genes to generate a low-dimensional representation of the data (Fig. 1*C*). PC analysis is a dimension reduction technique that extracts underlying structure of the data. It finds a sequence of linear combinations of the features/genes, as PCs, which have maximal variance. The first and second PCs (shown as PC1 and PC2 in Fig. 1*C*) are uncorrelated so that they can be uniquely estimated. Since TCR does not significantly contribute to the repair of the majority of (6-4)PPs in NHF1 cells, the first and second PCs do not differ between the TS and NTS repair. Importantly, a reconstructed repair trajectory lines up well with the time points, suggesting that repair pattern differs over the time course (Fig. 1*C*).

### Identification of repair super hotspots and super coldspots

We developed a computational framework to identify the early repair and late repair genomic sites by using time-course XR-seq data. Briefly, we first segmented the genome into consecutive bins of 50 bp long, then identified bins containing a significantly higher number of reads at early and late time points using a thresholding approach on the downsampled reads (Fig. S4). Figure 1*D* shows the distributions of read counts per genomic bin across all samples; we note enrichment of both early repair at 1 min and late repair at 4 h. In total, we identified 331 early repair genomic sites for (6-4)PP repair and 192 early repair genomic sites for CPD repair; we identified 105 late repair genomic sites for (6-4)PP repair (Tables S2–S4). These identified genomic sites are clusters of excision products, and we define the earliest-repair sites as repair super hotspots and the latest-repair sites as super coldspots. While this method was effective in identifying the top few hundred repair hotspots and coldspots, we also normalized and tested repair enrichment with a more rigorous Poisson log linear model (37, 38) on the read count data. We found that the identified repair super hotspots and super coldspots show enriched repair levels compared with those that would be expected under the null (Fig. S5) and are scattered across the entire human genome (Fig. S6).

**Figure 2:**
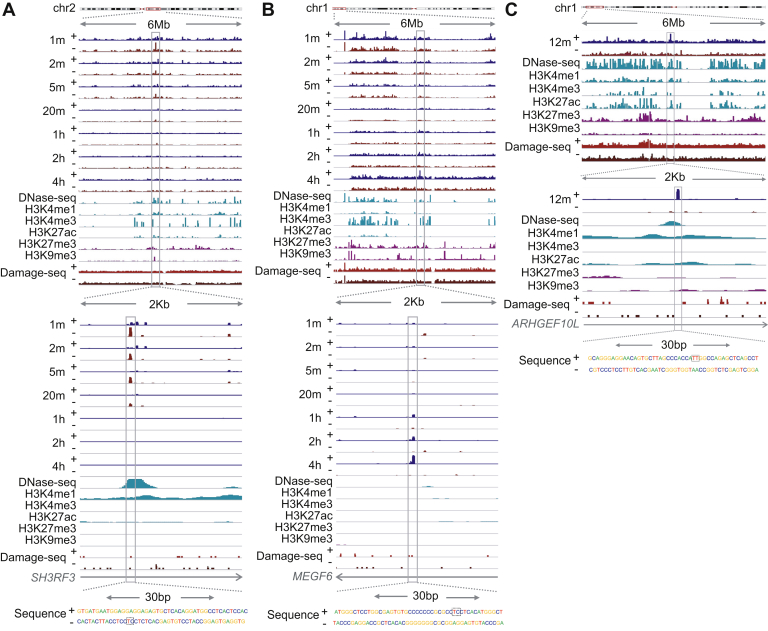
**Screenshots of representative repair super hotspots for (6-4)PP and CPD and representative repair super coldspots for (6-4)PP.** eXcision Repair-sequencing and Damage-seq data are shown for both strands, marked with + and −. Epigenetic data from ChIP-seq of histone modifications and DNase-seq are plotted on the same scale for cross-comparison. Read count data, normalized by sequencing depth, are visualized in the Integrative Genomics Viewer. *A*, a (6-4)PP repair super hotspot in chr2. *B*, a (6-4)PP repair super coldspot in chr1. *C*, a CPD repair super hotspot in chr1. All examples are from intronic gene regions overlapping annotated enhancers. Zoomed-in view of canonical sequences is overlaid in the *bottom*, with the damage sites shown in *dashed boxes*. (6-4)PP, (6-4) pyrimidine–pyrimidone photoproduct; CPD, cyclobutane pyrimidine dimer.

To gain further insight into the distribution of DNA damage, repair, and epigenomic markers around the identified repair super hotspots and super coldspots, we illustrate an example of each using screenshots. As shown in Figure 2, XR-seq signals from examples of repair super hotspots and super coldspots are separated by strand and plotted across all time points. We also include epigenomic signals from DNase-seq; ChIP-seq from ENCODE (39); and Damage-seq signals at 0 min after UV treatment (14). Specifically, the XR-seq signals from an example of a super hotspot for (6-4)PP repair decrease dramatically from 1 to 20 min, and they can be barely seen at 1 h (Fig. 2*A*). In contrast, the XR-seq signals at a super coldspot for (6-4)PP repair, shown in Figure 2*B*, increase over the time course and peak at 4 h. Another representative super hotspot for CPD repair is shown at 12 min (Fig. 2*C*). As can be seen in Figure 2, the size of the three representative spots is in the range of 50 bp.

### Neither repair super hotspots nor repair super coldspots overlap UV damage hotspots

As previously reported (12, 14, 29), the accessibility of repair machinery to the damage sites is a key factor affecting the repair rates of UV damage. In addition, it is reasonable to assume that genomic sites with high levels of damage are more likely to be subject to repair machinery than nearby sites with low levels of damage. A recent study assayed UV damage formation by adductSeq and freqSeq and reported a total of 157 hyper hotspots that acquired CPDs much more frequently than the genomic average in primary human fibroblasts (15). Of these CPD hyper hotspots, 83 are from the plus strand and 74 are from the minus strand, each with at least five recurrent sequence reads (15). To determine whether the identified repair super hotspots result simply from increased levels of UV damage, we first intersected the reported CPD hyper hotspots from Premi *et al.* (15) with the 192 CPD repair super hotspots that we identified; we found that none of our super hotspots overlap the reported CPD hyper hotspots.

To further confirm and replicate this seemingly striking result, we analyzed genome-wide CPD damage data generated by our previously developed Damage-seq protocol (14) in order to quantify damage levels at 0 time point after UV irradiation with single-nucleotide resolution. After stringent quality control procedures (refer to the Methods section for details), we identified 91 damage hotspots from the plus strand and 78 CPD damage hotspots from the minus strand, each with at least 10 mapped reads (Table S5). Notably, these CPD hotspots are shown to be enriched for heterochromatin and repressed regions (Fig. S7), which is concordant with previous reports (31, 40, 41). Again, none of the CPD hotspots identified from this parallel Damage-seq platform overlap the CPD repair super hotspots.

**Figure 3:**
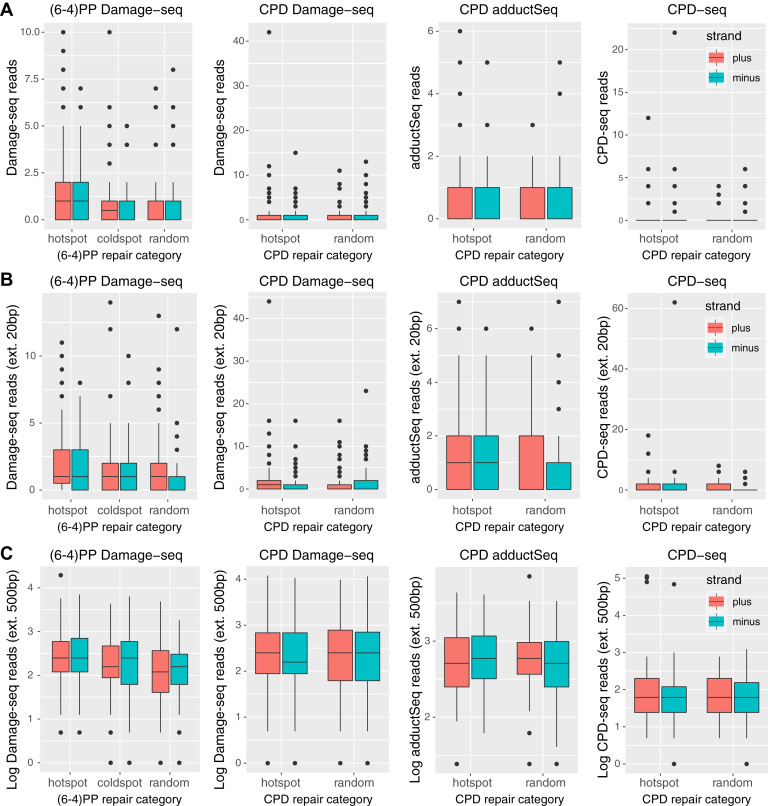
**Neither repair super hotspots nor super coldspots overlap hotspots of DNA damage.** There is no increase in DNA damage, as measured by Damage-seq, adductSeq, and CPD-seq, at the identified repair super hotspots for (6-4)PP and CPD. *A*, read counts for DNA damage are computed in repair super hotspots, super coldspots, and random spots. The regions corresponding to the different repair categories are extended at both ends for 20 bp (*B*) and 500 bp (*C*), respectively, to account for the shallow sequencing depth by quantifying DNA damage. (6-4)PP, (6-4) pyrimidine–pyrimidone photoproduct; CPD, cyclobutane pyrimidine dimer.

We also compared the DNA damage levels for (6-4)PP and CPD from three independent sequencing technologies—Damage-seq (14), adductSeq (15), and CPD-seq (25)—at our identified repair super hotspots and super coldspots against those from randomly sampled regions over the genome. To account for the sparse sampling when measuring DNA damage by NGS, we extend the regions corresponding to the repair super hotspots, super coldspots, and random spots at both ends for 20 and 500 bp, respectively. Our results, shown in Figure 3, suggest that there is no significant difference in the damage levels between the three repair categories (hotspot, coldspot, and random spot). The zoom-in and zoom-out views of three examples of repair super hotspots and super coldspots in Figure 2 also indicate that the distribution of Damage-seq reads is relatively uniform in the flanking regions. Previous results have demonstrated that UV-induced DNA damage is indeed virtually uniform across the entire human genome, whereas repair is affected by a variety of factors (such as chromatin states and transcription factor binding), depending on the type of DNA damage (14). While we note that the shallow depth of coverage of Damage-seq can be a limiting factor, our results validate our conclusion that the identified repair super hotspots and super coldspots are not damage formation hotspots.

### Repair super hotspots are enriched in FIREs and superenhancers

Early repair preferentially occurs in active and open chromatin regions because of the accessibility of repair machinery to damage sites (29, 42). Moreover, replication time is correlated with chromatin accessibility (43), and higher levels of excision repair have been observed in early replicating regions (44). It is not surprising that these hundreds of repair super hotspots are enriched in open chromatin regions and early replication domains. Indeed, we found that chromatin accessibility is higher for repair super hotspots and lower for super coldspots (Fig. S8); we also found an enrichment of repair super hotspots at promoters and enhancers (Figs. S9 and S10). When we intersected the identified repair super hotspots with the segmented replication domains from human fibroblast cell line IMR90 (43, 45), we found that these super hotspots are also significantly enriched in early replication domains (Fig. S11).

**Figure 4:**
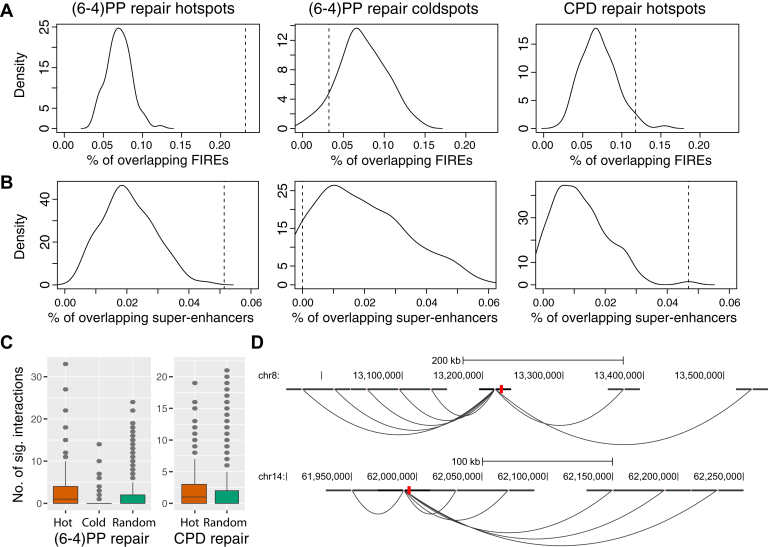
**Repair super hotspots are enriched in FIREs and superenhancers.** FIREs and superenhancers were identified and annotated using high-throughput chromosome conformation capture (Hi-C) data from human fibroblasts. Repair super hotspots overlap (*A*) FIREs and (*B*) superenhancers in significantly higher proportions than the genome-wide averages. The *solid density curves* are generated from bootstrapping different regions along the genome as the null case; the *dashed vertical lines* are the observed proportions for the repair super hotspots and super coldspots. *C*, repair super hotspots have a significantly higher number of significant interactions, identified by Hi-C. *D*, two examples of (6-4)PP repair super hotspots (chr8:13224201–13224300 and chr14:61994601–61994700) that overlap both FIREs and superenhancers and that loop to different regions of the genome. Hi-C data have low resolution, and the significant interactions are drawn from the center of each bin, which does not exactly overlap with the identified hotspot shown in *red*. (6-4)PP, (6-4) pyrimidine–pyrimidone photoproduct; CPD, cyclobutane pyrimidine dimer; FIREs, frequently interacting regions.

In the nucleus, the entire genomic DNA is hierarchically packaged to form a complex 3D genome architecture, which consists of multiscale structural units, including chromosome territories, A/B chromosomal compartments (46), topologically associating domains (TADs) (47), chromatin loops (48), long-range chromatin interactions (49), and FIREs (50). The 3D genome organization regulates a variety of cellular processes, such as transcription, DNA replication, and DNA damage formation and repair (51). It has been shown that DNA repair proteins bind at the boundary sites of chromosomally interacting domains in yeast cells, suggesting that this arrangement may promote the rapid repair of DNA damage in these regions (52). Despite recent progress in understanding UV susceptibility and repair efficiency in the context of genome architecture (31, 52), it is still unknown how 3D genome organization affects the excision repair of UV damage in humans. We therefore sought to determine how this architectural feature of 3D genome organization contributes to the identified repair super hotspots and super coldspots by using the publicly available high-throughput chromosome conformation capture (Hi-C) data from the human fibroblast cell line IMR90 (53, 54). Specifically, after quality control procedures and data normalization, we profiled FIREs using FIREcaller (50). After overlapping the repair super hotspots and super coldspots with the called FIREs (Table S6*A*), we found that a significantly higher proportion of repair super hotspots overlap FIREs—23.16% and 11.76% for (6-4)PP and CPD, respectively—compared with a genome average of 6.93% based on the profiled FIREs (Fig. 4*A*). Conversely, the overlapping proportion of (6-4)PP repair super coldspots is only 3.23%, significantly lower than the genome average (Fig. 4*A*).

FIREs have been previously reported to be enriched for superenhancers (55). We have demonstrated that the repair super hotspots are enriched in both FIREs and enhancers. We also observed that, across many cases, multiple enhancers that overlapped the repair super hotspots are from the same genomic regions (Fig. S12). As such, we expect that the repair super hotspots are also enriched in superenhancers, and we therefore adopted a list of previously annotated superenhancers in the human fibroblasts (56) (Table S6*B*). We found that, compared with a genome-wide average of 2.05%, the repair super hotspots are indeed enriched in superenhancers (5.14% and 4.69% for (6-4)PP and CPD repair hotspots, respectively), whereas none of the repair super coldspots overlap superenhancers (0% for (6-4)PP repair super coldspot) (Fig. 4*B*).

In addition, we detected significant interactions based on the Hi-C contact matrix using the Fit-Hi-C method (57) (Table S6*C*) and showed that repair super hotspots also overlap with a significantly higher number of significant interactions (Fig. 4*C*). Refer to the Methods section for details on data analysis. The overlapping information of the called repair super hotspots and super coldspots with the profiled FIREs, superenhancers, and significant chromatin interactions are included in Table S7. Figure 4*D* illustrates the loop interactions of two identified repair super hotspots. Notably, these two hotspots also overlap with both FIREs and superenhancers. Collectively, these results provide a global picture of genetic regulation of repair kinetics *via* 3D genome organization.

### Enrichment of cytosine in repair super hotspots and super coldspots

As mentioned, large-scale chromatin features such as replication timing and FIREs affect UV damage formation and repair. Local chromatin structure (*e.g.*, nucleosome and transcription factor binding) can also influence the distribution of UV damage formation and repair efficiency (30). Since the general size of our identified repair super hotspots and super coldspots is around 50 bp, we investigated the role of both local chromatin structure and large-scale chromatin features in these repair super hotspots and super coldspots.

**Figure 5:**
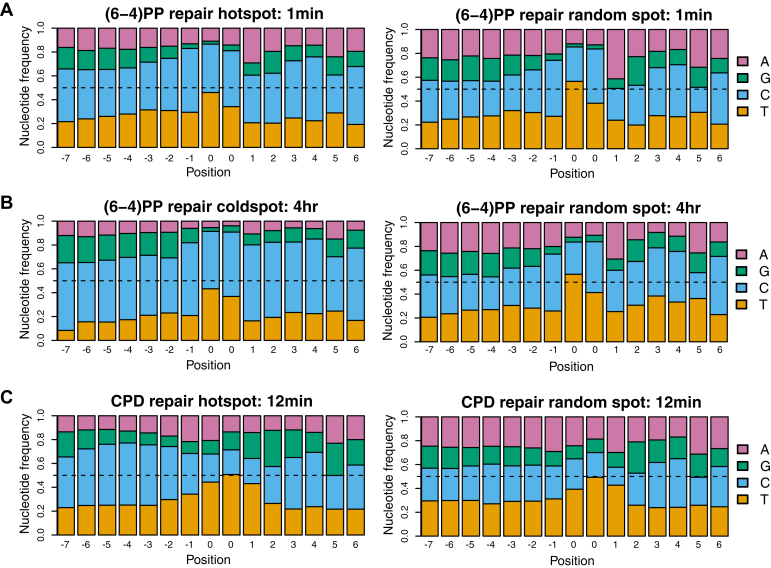
**Enrichment of cytosine in repair super hotspots and super coldspots.** Distributions of the nucleotide frequencies centered at the damage sites (marked as 0, 0) are shown. Frequencies are computed using all reads that are mapped to (*A*) (6-4)PP repair super hotspots, (*B*) (6-4)PP repair super coldspots, and (*C*) CPD repair super hotspots. As a comparison, the same number of excision repair reads are randomly sampled from the genome and used to generate the frequency distribution for the “random spot” in each panel. (6-4)PP, (6-4) pyrimidine–pyrimidone photoproduct; CPD, cyclobutane pyrimidine dimer.

To gain insight into how local chromatin structure contributes to the repair super hotspots and super coldspots, we performed sequence context analysis by using all reads mapped to the repair super hotspots and super coldspots, respectively. We trimmed the reads to 15 bp long, centering at the damage sites, and calculated strand-specific nucleotide frequencies in repair super hotspots, super coldspots, and randomly chosen spots. Interestingly, we identified an enrichment of cytosine in the flanking regions of the damage sites for both repair super hotspots and super coldspots (Fig. 5). We compared the cytosine frequency for repair super hotspots and super coldspots with that for the genomic bins used in this study. As shown in Fig. S13, the percentage of cytosine in both repair super hotspots and super coldspots is largely higher than that in the whole-genome regions.

Motif analysis by the MEME suite (58) confirmed the enrichment of cytosine adjacent to the damage sites, which are themselves enriched with canonical sequences of CTCA for (6-4)PP and TT for CPD (Table 1) (11). The predicted biological functions of these motifs include transcription-associated activities, such as regulation of transcription, sequence-specific DNA binding, transcription activator activity, and transcription factor activity. It has been previously shown that transcription factor binding can stimulate, inhibit, or produce no change on DNA damage formation depending on the nature of transcription factor and DNA-damaging agent (14, 59). Likewise, transcription factor binding can decrease or increase local repair activity and consequently affect the mutation rate in these binding regions (25, 27, 28, 33). Transcription factors (*e.g.*, Ets-1) have also been shown to interact with DNA repair machinery *in vivo* (60, 61). Thus, the identified repair super hotspots may be attributed to both local chromatin structures (*e.g.*, transcription factor binding or partially assembled repair machinery in the proximity of hotspots) and large-scale chromatin features (such as TADs and FIREs). The rapid removal of DNA damage in repair super hotspots in critical regions of the genome may aid cellular survival.

**Table 1:** Motif analysis of identified repair super hotspots and super coldspots

Category	Replicate	Sequence count	Motif	Motif sites	Top 3 predictions
(6-4 )PP repair hotspots	1	6984		6652	BP positive regulation of transcription from RNA polymerase II promoter BP anterior/posterior pattern formation MF calcium ion binding
	2	5863		5561	
(6-4)PP repair coldspots	1	1004		913	CC transcription factor complex MF potassium ion binding MF transcription activator activity
	2	990		908	
CPD repair hotspots	1	2268		1241	MF transcription factor activity MF sequence-specific DNA binding CC extracellular space

Motif analysis using the MEME suite (58) suggests enriched motifs with TC and TT dinucleotides at the damage sites. For (6-4)PP repair super hotspots and super coldspots, a canonical sequence of CTCA is enriched, as expected. For all three categories, there is enriched cytosine in the flanking regions. The gene ontology hierarchy is specified by the abbreviations (BP for biological process, CC for cellular component, and MF for molecular function) and the name of the term.

## Discussion

Chromatin features affect the distribution of DNA damage formation, repair efficiency, and subsequent mutational landscape (32, 33, 62). Knowing the exact genomic sites where the earliest-repair and latest-repair occur is critical for our understanding of the heterogeneity of DNA repair and mutation rate. We identified hundreds of repair super hotspots and super coldspots of UV damage that do not overlap with previously reported hotspots of UV damage and found that the repair super hotspots are enriched in FIREs (one of the features of 3D genome organization) and superenhancers. Furthermore, we discovered an enrichment of cytosine in areas flanking the damage sites in both the repair super hotspots and super cspots. This unique sequence context might be the target DNA sequence for binding of transcription factors that increase or decrease the damage formation and repair activity. The aforementioned local chromatin structures, as well as large-scale chromatin features, may therefore explain the formation of repair super hotspots and super coldspots.

Deciphering the interplay between DNA damage formation and repair efficiency is also crucial for the study of mutation distribution. Although a variety of NGS-based methods have been developed to detect DNA damage formation and repair over the last 5 years (9), XR-seq, to the best of our knowledge, is the only method that can be used to determine the genome-wide repair super hotspots and super coldspots. In comparison with other approaches that measure repair indirectly by subtracting two large percentages of damage, XR-seq directly detects excision repair events with virtually no background noise. In this study, we managed to perform XR-seq as early as 1 min after UV irradiation, making it possible to detect the earliest repaired genomic sites; these sites could not be identified even at a 5-min time point in our previous study (29). With respect to the distribution of DNA damage formation, our previous results showed a uniform damage distribution pattern across the whole genome (14). However, because of the low coverage depth of Damage-seq, the uniform pattern can be observed only at a large scale (*e.g.*, mega base), not at a small scale (*e.g.*, kilobase). Both the sequencing depth and the scale are two important factors that we must consider when we interpret the distribution pattern of DNA damage. The damage formation hotspots used in this study may be underrepresented because of the low coverage depth, despite the computational approaches applied to identify hotspots of DNA damage formation using multiomics datasets.

How and why cells prioritize these super hotspots for rapid repair but leave damage in super coldspots until the repair is almost complete is not completely understood at present. Here, our findings suggest that both large-scale chromatin features and local chromatin structures may determine the order of repair. This triage-like mechanism would allow cells to prioritize DNA damage removal based on their location in the genome. This type of triage takes place in other contexts. For example, it has been shown that the lamina-associated heterochromatin is more vulnerable than active euchromatin (31), and DNA repair factors (*e.g.*, BRCA1) were discovered to bind to highly interacting regions within chromosomes (63). In response to ionizing radiation, cells exhibit an increased segregation of TADs, which may play a protective role against DNA damage (64). We found that repair super hotspots are enriched in FIREs (23.26% and 11.76% for (6-4)PP and CPD, respectively), whereas only 3.23% of (6-4)PP repair super coldspots overlap FIREs. The rapid removal of DNA damage in these active genomic regions such as FIREs may aid in cellular survival.

There may be several ways in which local chromatin structure contributes to the origin of repair super hotspots and super coldspots, including the binding of transcription factor, DNA sequence context, and the presence of partially preassembled DNA repair machinery. Some transcription factors (*e.g.*, tryptophan cluster factors) have a stimulatory effect on UV damage formation (25, 28), whereas other factors (*e.g.*, SP1) have an inhibitory effect (14). This may be explained by the different levels of DNA conformational changes caused by the binding of different transcription factors; these changes may make the local DNA sequence more or less vulnerable to DNA-damaging agents (65).

In addition to the effect of transcription factor binding, the local sequence context itself, which shows cytosine enrichment in the areas flanking the damage sites, may be vulnerable to UV damage. Although we found that the observed repair super hotspots and super coldspots are not DNA damage hotspots, they can register high damage levels upon UV irradiation. The presence of preassembled DNA repair machinery at specific genomic regions likely also promotes repaid repair of DNA damage in these regions. Indeed, global repair complex was found to bind at chromosomally interacting domain boundaries in the absence of DNA damage; this preassembled DNA repair complex will initiate efficient repair at these regions (52). Moreover, most excision repair proteins are known to be involved in other genomic transactions: Transcription factor II H (TFIIH), an important protein complex, is both a general transcription factor for RNA polymerase II and an essential component of nucleotide excision repair complex (66); XPC, in complex with RAD23B, also functions in transcription (67); and XPG and XPF, two nucleases for dual incision in nucleotide excision repair, are also required to form chromatin looping through recruitment of the CCCTC-binding factor (68). In addition, it has been reported that EST1 interacts with DNA-dependent protein kinase and poly (ADP-ribose) polymerase-1 (60, 69).

Given this evidence, it is reasonable to propose the following model for a mechanism underlying the origin of repair super hotspots identified in this study: In active chromatin regions, stimulatory transcription factors bind to the repair super hotspots with unique sequence contexts that are vulnerable to UV radiation. Meanwhile, their interacting partners, the excision repair machinery, are positioned in close proximity to the super hotspots. Upon UV irradiation, higher levels of DNA damage are produced in repair super hotspots than in their adjacent regions. Immediately, the preassembled excision repair machinery will recognize and remove the damage through nucleotide excision repair. In this way, cells protect gene expression and survive the external stress of DNA damage. Conversely, in the case of repair super coldspots, the inaccessibility of the chromatin region and the sequence context's vulnerability to UV may explain why damage at these sites is not removed until repair of the entire genome is almost complete.

Collectively, our results identify repair super hotspots and super coldspots of UV damage in the human genome, which may be attributed to large-scale chromatin features and local chromatin structures. We believe that the methodology and data presented in this article will aid in future research on DNA damage, repair, mutagenesis, and carcinogenesis.

## Experimental procedures

### Cell culture and UV irradiation

Human NHF1 cells were obtained from W. K. Kaufmann (University of North Carolina, Chapel Hill) (70) and cultured in Dulbecco's modified Eagle's medium with 10% fetal bovine serum at 37 °C in a 5% CO_2_ humidified chamber. For (6-4)PP XR-seq at 1 and 2 min time points, UV irradiation was performed as previously described (11, 13). Briefly, the 80% confluent NHF1 cells in one Petri dish were irradiated for 20 s under a 250 nm UV lamp (1 J/m^2^/s) after removing the culture medium. Dulbecco's modified Eagle's medium with 10% fetal bovine serum medium at 37 °C was immediately added into the Petri dish, then the medium was poured off, and the Petri dish was put on ice promptly at the end of 1 min or 2 min after UV irradiation. The time count starts from the end of 20 s UV irradiation and ends at the time point when the Petri dish is put on ice. The cells were washed one time with ice-cold PBS before being harvested by a cell scraper in 10 ml ice-cold PBS. In each replicate of (6-4)PP XR-seq experiment, 50 and 30 Petri dishes (150 × 15 mm) containing NHF1 cells were treated one by one at 1 and 2 min time points, respectively. Cell culture, UV treatment, and library preparation for (6-4)PP XR-seq at 5 min, 20 min, 1 h, 2 h, and 4 h and CPD XR-seq at 12 min were performed in previous studies (11, 22, 29). For *in vivo* excision assay, UV irradiation was performed as aforementioned, and 10 and 5 Petri dishes (150 × 15 mm) containing NHF1 cells were used at 0 and 2 min time points, respectively.

### Excision assay

The *in vivo* excision assay was performed as described (17, 34). Following UV irradiation, the excision products were isolated by gentle cell lysis and nonchromatin fraction separation and purified by TFIIH immunoprecipitation. The purified excision products were then 3’ radiolabeled by terminal deoxynucleotidyl transferase and [α-^32^P]-3’-dATP and resolved in a 10% denaturing acrylamide gel. Ten and five Petri dishes (150 × 15 mm) of NHF1 cells were used at 0 and 2 min, respectively.

### XR-seq library preparation and sequencing

XR-seq libraries were prepared as described in the previous protocol (17). Briefly, the excision products were isolated by TFIIH immunoprecipitation following gentle cell lysis and nonchromatin fraction separation and ligated with adaptors. The ligated excision products were then further purified by immunoprecipitation with anti-(6-4)PP antibody and repaired by (6-4)PP photolyase before the library amplification by PCR. Libraries were sequenced on an Illumina HiSeq 4000 platform.

### Data collection

(6-4)PP XR-seq data at 5 min, 20 min, 1 h, 2 h, and 4 h were downloaded from the Gene Expression Omnibus (GEO) with accession numbers GSE67941 (11) and GSE76391 (29). CPD XR-seq data at 12 min were downloaded from GEO with accession number GSE138846 (22). CPD and (6-4)PP damage data of NHF1 by Damage-seq were downloaded from GEO with accession number GSE98025 (14); CPD damage data of NHF1 by CPD-seq were downloaded from GEO with accession number GSM2772322 and GSM2772323 (25); CPD damage data of human primary fibroblast by adductSeq were downloaded from GEO with accession number GSM4073616 and GSM4073634 (15). Hyper hotspots for UV-induced CPD damage in primary human fibroblasts were downloaded from the study by Premi *et al.* (15). Normal human dermal fibroblasts H3K4me1 (ENCODE Data Coordination Center accession number: ENCSR000ARV), H3K4me3 (accession number: ENCSR000DPR), H3K27ac (accession number: ENCSR000APN), H3K27me3 (accession number: ENCSR000APO), H3K9me3 (accession number: ENCSR000ARX), and DNase-seq (accession number: ENCSR000EMP) data were downloaded from the ENCODE portal (39). Normal human lung fibroblasts chromatin state segmentation results by ChromHMM were downloaded from University of California Santa Cruz accession number wgEncodeEH000792 (71). Hi-C data of IMR90 were downloaded from GEO with accession number GSE43070 (53) and from https://bioconductor.org/packages/HiCDataHumanIMR90/ (54). The list of annotated superenhancers in IMR90 was downloaded from the Roadmap Epigenomics Consortium (56). Genomic categories of replication timing from Repli-Seq data of IMR90 (43) were downloaded from GSE53984 (45).

### Bioinformatics and statistical analysis

#### XR-seq bioinformatics preprocessing

For XR-seq, Cutadapt (72) was used to trim reads with adaptor sequence TGGAATTCTCGGGTGCCAAGGAACTCCAGTNNNNNNACGATCTCGTATGCCGTCTTCTGCTTG at the 3’ end and discard untrimmed reads. Burrows-Wheeler Aligner (73) was used for alignment of single-end short reads. Unmapped reads and reads that map to multiple locations with the same alignment quality were removed using Samtools (bioinformatics tools written in C for manipulating NGS data) (74). Postalignment filtering steps were adopted using Rsamtools (http://bioconductor.org/packages/Rsamtools/). Specifically, if multiple reads share the same 5’ and 3’ end coordinates, we keep only one to perform deduplication. We also only keep reads that have mapping quality greater than 20 and are of lengths 21 to 31 bp.

#### Gene-level quantification of excision repair

Reads from the TS and NTS strands were separated using known gene annotations for the human genome assembly hg19 by ENSEMBL. We use reads per kilobase per million mapped reads for within-sample normalization for the XR-seq data. To perform gene-level quantification and downstream analysis including segmented regression, we adopted a stringent quality control procedure and only retained genes that (i) had at least 10 TT or TC dinucleotides from either TS or NTS; (ii) were less than 300 kb; and (iii) had at least ten reads in total across all XR-seq samples. In addition, we took the ratio of the reads from the TS and NTS (TS/[TS + NTS]) to remove biases and artifact that are shared between the two DNA strands, that is, library size, gene length, and other gene-specific biases, such as sequencing bias and antibody pull-down efficiency, and others. The ratio is bound between 0 and 1 and sheds light upon how TCR and global repair interplay (Fig. S3).

#### Identification of repair super hotspots and super coldspots

We started by segmenting the human reference genome into consecutive bins of 50 bp long. We then calculated the observed depth of coverage per bin by XR-seq, separating the plus-strand reads (+) and minus-strand reads (−). To mitigate the effect of library size/sequencing depth, we downsampled the reads in each sample to 7.7 million without replacement. To identify repair super hotspots and super coldspots, we set a threshold on the number of read counts per genomic bin in the 1 min and 4 h samples. Specifically, to identify (6-4)PP repair super hotspots, we require at least 15 reads mapped in both replicates at 1 min and at most five reads mapped in both replicates at 4 h. The read count threshold is relaxed for the identification of super coldspots, which have a smaller number compared with the super hotspots. For CPD repair, to avoid complications because of TCR at later time points, we focused on CPD repair super hotspots only.

In addition to the thresholding approach, we adopted a more rigorous cross-sample Poisson log linear model (37, 38) for data normalization. Specifically, we denote *Y* as the observed repair matrix, with row *i* corresponding to the *i*th genomic bin and column *j* corresponding to the *j*th sample. The “null” model, which reflects the expected coverage when there is no biologically relevant repair enrichment, is

$$Y_{ij}∼\text{Poisson}\left(\lambda_{ij}\right),\lambda_{ij}=N_{j}\beta_{i}f_{j}\left(TC_{i}\right),$$

where *N*_*j*_ is the total number of mapped reads for sample *j* (fixed for downsampled data), β_*i*_ reflects the bin-specific bias because of library preparation and sequencing bias, and *f*_*j*_(*TC*_*i*_) is the sample-specific bias because of TC (thymine and cytosine) content for damage/repair. The goal of fitting the null model to the data is to estimate the various sources of biases, which can then be used for normalization. We adopt a robust iterative maximum-likelihood algorithm (38) for estimating the parameters of the null model. Plus and minus strands are analyzed separately.

Given a first pass of the calling algorithms, we identified strong repair super hotspots in pericentromeric regions, which were collapsed repeats annotated as unique sequences in the reference genome (*e.g.*, ribosomal DNA (21)). It is important to exclude artifacts as stringently as possible, and thus we undertook an additional quality control step. “Blacklist” bins, including segmental duplication regions (http://humanparalogy.gs.washington.edu/build37/data/GRCh37GenomicSuperDup.tab), gaps in reference assembly from telomere, centromere, and/or heterochromatin regions (https://gist.github.com/leipzig/6123703), and repeating elements by RepeatMasker (https://genome.ucsc.edu/cgi-bin/hgTrackUi?g=rmsk) are masked in downstream analysis.

#### Hi-C data analysis

We adopted the Hi-C data of human fibroblast cell line IMR90 (53, 54) to investigate the relationship between identified repair super hotspots and the 3D genome organization. We took the raw contact matrix with 40 kb resolution as input and detected FIREs, which play important roles in transcriptional regulations, across the entire genome using FIREcaller (50). To further investigate whether these repair super hotspots are involved in functional chromatin looping between regulatory elements and their target genes, we adopted the Fit-Hi-C approach (57) to identify long-range chromatin interactions on all 40 kb bin pairs within a maximal 3 MB region. The interactions with *p* value <2.31e-11 were considered as statistically significant (75).

## Data and code availability

The data reported in this article have been deposited in GEO with accession number GSE148303, and all remaining data are contained within the article. Scripts used in this article are available at https://github.com/yuchaojiang/damage_repair.

## Supporting information

This article contains supporting information (40, 41, 50, 53, 55, 75, 76).

## Conflict of interest

The authors declare that they have no conflicts of interest with the contents of this article.

## References

[1] Wood R.D. (1997). Nucleotide excision repair in mammalian cells. J. Biol. Chem..

[2] Sancar A. (2016). Mechanisms of DNA repair by photolyase and excision nuclease (nobel lecture). Angew. Chem. Int. Ed. Engl..

[3] Mellon I., Spivak G., Hanawalt P.C. (1987). Selective removal of transcription-blocking DNA damage from the transcribed strand of the mammalian DHFR gene. Cell.

[4] Hanawalt P.C., Spivak G. (2008). Transcription-coupled DNA repair: Two decades of progress and surprises. Nat. Rev..

[5] Reardon J.T., Sancar A. (2005). Nucleotide excision repair. Prog. Nucleic Acid Res. Mol. Biol..

[6] Hu J., Selby C.P., Adar S., Adebali O., Sancar A. (2017). Molecular mechanisms and genomic maps of DNA excision repair in Escherichia coli and humans. J. Biol. Chem..

[7] Hodis E., Watson I.R., Kryukov G.V., Arold S.T., Imielinski M., Theurillat J.P., Nickerson E., Auclair D., Li L., Place C., Dicara D., Ramos A.H., Lawrence M.S., Cibulskis K., Sivachenko A. (2012). A landscape of driver mutations in melanoma. Cell.

[8] Martincorena I., Roshan A., Gerstung M., Ellis P., Van Loo P., McLaren S., Wedge D.C., Fullam A., Alexandrov L.B., Tubio J.M., Stebbings L., Menzies A., Widaa S., Stratton M.R., Jones P.H. (2015). Tumor evolution. High burden and pervasive positive selection of somatic mutations in normal human skin. Science.

[9] Li W., Sancar A. (2020). Methodologies for detecting environmentally induced DNA damage and repair. Environ. Mol. Mutagen..

[10] Bryan D.S., Ransom M., Adane B., York K., Hesselberth J.R. (2014). High resolution mapping of modified DNA nucleobases using excision repair enzymes. Genome Res..

[11] Hu J., Adar S., Selby C.P., Lieb J.D., Sancar A. (2015). Genome-wide analysis of human global and transcription-coupled excision repair of UV damage at single-nucleotide resolution. Genes Dev..

[12] Mao P., Smerdon M.J., Roberts S.A., Wyrick J.J. (2016). Chromosomal landscape of UV damage formation and repair at single-nucleotide resolution. Proc. Natl. Acad. Sci. U. S. A..

[13] Li W., Hu J., Adebali O., Adar S., Yang Y., Chiou Y.Y., Sancar A. (2017). Human genome-wide repair map of DNA damage caused by the cigarette smoke carcinogen benzo[a]pyrene. Proc. Natl. Acad. Sci. U. S. A..

[14] Hu J., Adebali O., Adar S., Sancar A. (2017). Dynamic maps of UV damage formation and repair for the human genome. Proc. Natl. Acad. Sci. U. S. A..

[15] Premi S., Han L., Mehta S., Knight J., Zhao D., Palmatier M.A., Kornacker K., Brash D.E. (2019). Genomic sites hypersensitive to ultraviolet radiation. Proc. Natl. Acad. Sci. U. S. A..

[16] Hu J., Lieb J.D., Sancar A., Adar S. (2016). Cisplatin DNA damage and repair maps of the human genome at single-nucleotide resolution. Proc. Natl. Acad. Sci. U. S. A..

[17] Hu J., Li W., Adebali O., Yang Y., Oztas O., Selby C.P., Sancar A. (2019). Genome-wide mapping of nucleotide excision repair with XR-seq. Nat. Protoc..

[18] Adebali O., Chiou Y.Y., Hu J., Sancar A., Selby C.P. (2017). Genome-wide transcription-coupled repair in Escherichia coli is mediated by the Mfd translocase. Proc. Natl. Acad. Sci. U. S. A..

[19] Li W., Adebali O., Yang Y., Selby C.P., Sancar A. (2018). Single-nucleotide resolution dynamic repair maps of UV damage in Saccharomyces cerevisiae genome. Proc. Natl. Acad. Sci. U. S. A..

[20] Oztas O., Selby C.P., Sancar A., Adebali O. (2018). Genome-wide excision repair in Arabidopsis is coupled to transcription and reflects circadian gene expression patterns. Nat. Commun..

[21] Yang Y., Hu J., Selby C.P., Li W., Yimit A., Jiang Y., Sancar A. (2019). Single-nucleotide resolution analysis of nucleotide excision repair of ribosomal DNA in humans and mice. J. Biol. Chem..

[22] Deger N., Yang Y., Lindsey-Boltz L.A., Sancar A., Selby C.P. (2019). Drosophila, which lacks canonical transcription-coupled repair proteins, performs transcription-coupled repair. J. Biol. Chem..

[23] Selby C.P., Lindsey-Boltz L.A., Yang Y., Sancar A. (2020). Mycobacteria excise DNA damage in 12- or 13-nucleotide-long oligomers by prokaryotic-type dual incisions and performs transcription-coupled repair. J. Biol. Chem..

[24] Akkose U., Kaya V.O., Lindsey-Boltz L., Karagoz Z., Brown A.D., Larsen P.A., Yoder A.D., Sancar A., Adebali O. (2020). Comparative analyses of two primate species diverged by more than 60 million years show different rates but similar distribution of genome-wide UV repair events. bioRxiv.

[25] Mao P., Brown A.J., Esaki S., Lockwood S., Poon G.M.K., Smerdon M.J., Roberts S.A., Wyrick J.J. (2018). ETS transcription factors induce a unique UV damage signature that drives recurrent mutagenesis in melanoma. Nat. Commun..

[26] Poulos R.C., Thoms J.A.I., Guan Y.F., Unnikrishnan A., Pimanda J.E., Wong J.W.H. (2016). Functional mutations form at CTCF-cohesin binding sites in melanoma due to uneven nucleotide excision repair across the motif. Cell Rep..

[27] Sabarinathan R., Mularoni L., Deu-Pons J., Gonzalez-Perez A., Lopez-Bigas N. (2016). Nucleotide excision repair is impaired by binding of transcription factors to DNA. Nature.

[28] Frigola J., Sabarinathan R., Gonzalez-Perez A., Lopez-Bigas N. (2020). Variable interplay of UV-induced DNA damage and repair at transcription factor binding sites. Nucleic Acids Res..

[29] Adar S., Hu J., Lieb J.D., Sancar A. (2016). Genome-wide kinetics of DNA excision repair in relation to chromatin state and mutagenesis. Proc. Natl. Acad. Sci. U. S. A..

[30] Mao P., Wyrick J.J., Roberts S.A., Smerdon M.J. (2017). UV-induced DNA damage and mutagenesis in chromatin. Photochem. Photobiol..

[31] Garcia-Nieto P.E., Schwartz E.K., King D.A., Paulsen J., Collas P., Herrera R.E., Morrison A.J. (2017). Carcinogen susceptibility is regulated by genome architecture and predicts cancer mutagenesis. EMBO J..

[32] Roberts S.A., Brown A.J., Wyrick J.J. (2019). Recurrent noncoding mutations in skin cancers: UV damage susceptibility or repair inhibition as primary driver?. Bioessays.

[33] Perera D., Poulos R.C., Shah A., Beck D., Pimanda J.E., Wong J.W. (2016). Differential DNA repair underlies mutation hotspots at active promoters in cancer genomes. Nature.

[34] Hu J., Choi J.H., Gaddameedhi S., Kemp M.G., Reardon J.T., Sancar A. (2013). Nucleotide excision repair in human cells: Fate of the excised oligonucleotide carrying DNA damage *in vivo*. J. Biol. Chem..

[35] Li W., Liu W., Kakoki A., Wang R., Adebali O., Jiang Y., Sancar A. (2019). Nucleotide excision repair capacity increases during differentiation of human embryonic carcinoma cells into neurons and muscle cells. J. Biol. Chem..

[36] Jolliffe I.T., Cadima J. (2016). Principal component analysis: A review and recent developments. Philos. Trans. A Math. Phys. Eng. Sci..

[37] Witten D.M. (2011). Classification and clustering of sequencing data using a Poisson model. Ann. Appl. Stat..

[38] Jiang Y.C., Oldridge D.A., Diskin S.J., Zhang N.R. (2015). CODEX: A normalization and copy number variation detection method for whole exome sequencing. Nucleic Acids Res..

[39] ENCODE Project Consortium (2012). An integrated encyclopedia of DNA elements in the human genome. Nature.

[40] Han C., Srivastava A.K., Cui T., Wang Q.E., Wani A.A. (2016). Differential DNA lesion formation and repair in heterochromatin and euchromatin. Carcinogenesis.

[41] Hauer M.H., Gasser S.M. (2017). Chromatin and nucleosome dynamics in DNA damage and repair. Genes Dev..

[42] Yimit A., Adebali O., Sancar A., Jiang Y. (2019). Differential damage and repair of DNA-adducts induced by anti-cancer drug cisplatin across mouse organs. Nat. Commun..

[43] Hansen R.S., Thomas S., Sandstrom R., Canfield T.K., Thurman R.E., Weaver M., Dorschner M.O., Gartler S.M., Stamatoyannopoulos J.A. (2010). Sequencing newly replicated DNA reveals widespread plasticity in human replication timing. Proc. Natl. Acad. Sci. U. S. A..

[44] Poulos R.C., Olivier J., Wong J.W.H. (2017). The interaction between cytosine methylation and processes of DNA replication and repair shape the mutational landscape of cancer genomes. Nucleic Acids Res..

[45] Liu F., Ren C., Li H., Zhou P., Bo X., Shu W. (2016). De novo identification of replication-timing domains in the human genome by deep learning. Bioinformatics.

[46] Lieberman-Aiden E., van Berkum N.L., Williams L., Imakaev M., Ragoczy T., Telling A., Amit I., Lajoie B.R., Sabo P.J., Dorschner M.O., Sandstrom R., Bernstein B., Bender M.A., Groudine M., Gnirke A. (2009). Comprehensive mapping of long-range interactions reveals folding principles of the human genome. Science.

[47] Dixon J.R., Gorkin D.U., Ren B. (2016). Chromatin domains: The unit of chromosome organization. Mol. Cell.

[48] Rao S.S., Huntley M.H., Durand N.C., Stamenova E.K., Bochkov I.D., Robinson J.T., Sanborn A.L., Machol I., Omer A.D., Lander E.S., Aiden E.L. (2014). A 3D map of the human genome at kilobase resolution reveals principles of chromatin looping. Cell.

[49] Kaul A., Bhattacharyya S., Ay F. (2020). Identifying statistically significant chromatin contacts from Hi-C data with FitHiC2. Nat. Protoc..

[50] Crowley C., Yang Y., Qiu Y., Hu B., Abnousi A., Lipinski J., Plewczynski D., Wu D., Won H., Ren B., Hu M., Li Y. (2020). FIREcaller: Detecting frequently interacting regions from Hi-C data. bioRxiv.

[51] Zheng H., Xie W. (2019). The role of 3D genome organization in development and cell differentiation. Nat. Rev..

[52] van Eijk P., Nandi S.P., Yu S., Bennett M., Leadbitter M., Teng Y., Reed S.H. (2019). Nucleosome remodeling at origins of global genome-nucleotide excision repair occurs at the boundaries of higher-order chromatin structure. Genome Res..

[53] Jin F., Li Y., Dixon J.R., Selvaraj S., Ye Z., Lee A.Y., Yen C.A., Schmitt A.D., Espinoza C.A., Ren B. (2013). A high-resolution map of the three-dimensional chromatin interactome in human cells. Nature.

[54] Dixon J.R., Selvaraj S., Yue F., Kim A., Li Y., Shen Y., Hu M., Liu J.S., Ren B. (2012). Topological domains in mammalian genomes identified by analysis of chromatin interactions. Nature.

[55] Schmitt A.D., Hu M., Jung I., Xu Z., Qiu Y., Tan C.L., Li Y., Lin S., Lin Y., Barr C.L., Ren B. (2016). A compendium of chromatin contact maps reveals spatially active regions in the human genome. Cell Rep..

[56] Kundaje A., Meuleman W., Ernst J., Bilenky M., Yen A., Heravi-Moussavi A., Kheradpour P., Zhang Z., Wang J., Ziller M.J., Amin V., Whitaker J.W., Schultz M.D., Ward L.D., Roadmap Epigenomics Consortium (2015). Integrative analysis of 111 reference human epigenomes. Nature.

[57] Ay F., Bailey T.L., Noble W.S. (2014). Statistical confidence estimation for Hi-C data reveals regulatory chromatin contacts. Genome Res..

[58] Bailey T.L., Boden M., Buske F.A., Frith M., Grant C.E., Clementi L., Ren J., Li W.W., Noble W.S. (2009). Meme suite: Tools for motif discovery and searching. Nucleic Acids Res..

[59] Elliott K., Bostrom M., Filges S., Lindberg M., Van den Eynden J., Stahlberg A., Clausen A.R., Larsson E. (2018). Elevated pyrimidine dimer formation at distinct genomic bases underlies promoter mutation hotspots in UV-exposed cancers. PLoS Genet..

[60] Legrand A.J., Choul-Li S., Spriet C., Idziorek T., Vicogne D., Drobecq H., Dantzer F., Villeret V., Aumercier M. (2013). The level of Ets-1 protein is regulated by poly(ADP-ribose) polymerase-1 (PARP-1) in cancer cells to prevent DNA damage. PLoS One.

[61] Choul-Li S., Legrand A.J., Bidon B., Vicogne D., Villeret V., Aumercier M. (2018). Ets-1 interacts through a similar binding interface with Ku70 and poly (ADP-ribose) polymerase-1. Biosci. Biotechnol. Biochem..

[62] Gonzalez-Perez A., Sabarinathan R., Lopez-Bigas N. (2019). Local determinants of the mutational landscape of the human genome. Cell.

[63] Sobhy H., Kumar R., Lewerentz J., Lizana L., Stenberg P. (2019). Highly interacting regions of the human genome are enriched with enhancers and bound by DNA repair proteins. Sci. Rep..

[64] Sanders J.T., Freeman T.F., Xu Y., Golloshi R., Stallard M.A., Hill A.M., San Martin R., Balajee A.S., McCord R.P. (2020). Radiation-induced DNA damage and repair effects on 3D genome organization. Nat. Commun..

[65] Tornaletti S., Pfeifer G.P. (1995). UV light as a footprinting agent: Modulation of UV-induced DNA damage by transcription factors bound at the promoters of three human genes. J. Mol. Biol..

[66] Drapkin R., Reardon J.T., Ansari A., Huang J.C., Zawel L., Ahn K., Sancar A., Reinberg D. (1994). Dual role of TFIIH in DNA excision repair and in transcription by RNA polymerase II. Nature.

[67] Fong Y.W., Cattoglio C., Tjian R. (2013). The intertwined roles of transcription and repair proteins. Mol. Cell.

[68] Le May N., Fradin D., Iltis I., Bougneres P., Egly J.M. (2012). XPG and XPF endonucleases trigger chromatin looping and DNA demethylation for accurate expression of activated genes. Mol. Cell.

[69] Choul-li S., Drobecq H., Aumercier M. (2009). DNA-dependent protein kinase is a novel interaction partner for Ets-1 isoforms. Biochem. Biophys. Res. Commun..

[70] Heffernan T.P., Simpson D.A., Frank A.R., Heinloth A.N., Paules R.S., Cordeiro-Stone M., Kaufmann W.K. (2002). An ATR- and Chk1-dependent S checkpoint inhibits replicon initiation following UVC-induced DNA damage. Mol. Cell. Biol..

[71] Ernst J., Kellis M. (2012). ChromHMM: Automating chromatin-state discovery and characterization. Nat. Methods.

[72] Martin M. (2011). Cutadapt removes adapter sequences from high-throughput sequencing reads. EMBNET J..

[73] Li H., Durbin R. (2009). Fast and accurate short read alignment with Burrows-Wheeler transform. Bioinformatics.

[74] Li H., Handsaker B., Wysoker A., Fennell T., Ruan J., Homer N., Marth G., Abecasis G., Durbin R., 1000 Genome Project Data Processing Subgroup (2009). The sequence alignment/map format and SAMtools. Bioinformatics.

[75] Giusti-Rodríguez P., Lu L., Yang Y., Crowley C.A., Liu X., Juric I., Martin J.S., Abnousi A., Allred S.C., Ancalade N. (2019). Using three-dimensional regulatory chromatin interactions from adult and fetal cortex to interpret genetic results for psychiatric disorders and cognitive traits. bioRxiv.

[76] Wolfe D., Dudek S., Ritchie M.D., Pendergrass S.A. (2013). Visualizing genomic information across chromosomes with PhenoGram. BioData Min..

