# Supplementary material for: Super hotspots and super coldspots in the repair of UV-induced DNA damage in the human genome
Source: J Biol Chem. 2021 Mar 23;296:100581. doi: 10.1016/j.jbc.2021.100581 (PMC8081918; doi:10.1016/j.jbc.2021.100581)
Supplement: Supplemental Figures S1–S13 and Table S1 [file mmc3.pdf]

Supplements to "Super-hotspots and -coldspots in the repair of UV-induced DNA damage in the human genome"

Yuchao Jiang[1,2,3,*,#], Wentao Li[4,*,&], Laura A Lindsey-Boltz[4,*], Yuchen Yang[2], Yun Li[1,2,5], Aziz Sancar[3,4,#]

1 Department of Biostatistics, Gillings School of Global Public Health, University of North Carolina, Chapel Hill, NC 27599, USA.

2 Department of Genetics, School of Medicine, University of North Carolina, Chapel Hill, NC 27599, USA.

3 Lineberger Comprehensive Cancer Center, University of North Carolina, Chapel Hill, NC 27599, USA.

4 Department of Biochemistry and Biophysics, School of Medicine, University of North Carolina, Chapel Hill, NC 27599, USA.

5 Department of Computer Science, College of Arts and Sciences, University of North Carolina, Chapel Hill, NC 27599, USA.

* These authors contributed equally.

# To whom correspondence should be addressed. Email: yuchaoj@email.unc.edu, aziz_sancar@med.unc.edu.

& Present address: Department of Environmental Health Science, College of Public Health, University of Georgia, Athens, GA 30602, USA.

Figure S1: XR-seq signals between two biological replicates. (6-4)PP DNA repair levels between each pair of biological replicates collected at (A) 1 min, (B) 2 min, (C) 5 min, (D) 20 min, (E) 1 h, (F) 2 h, and (G) 4 h. The two (6-4)PP XR-seq replicates across all measured timepoints have correlation coefficients above 0.99, indicating good data quality and reproducibility.

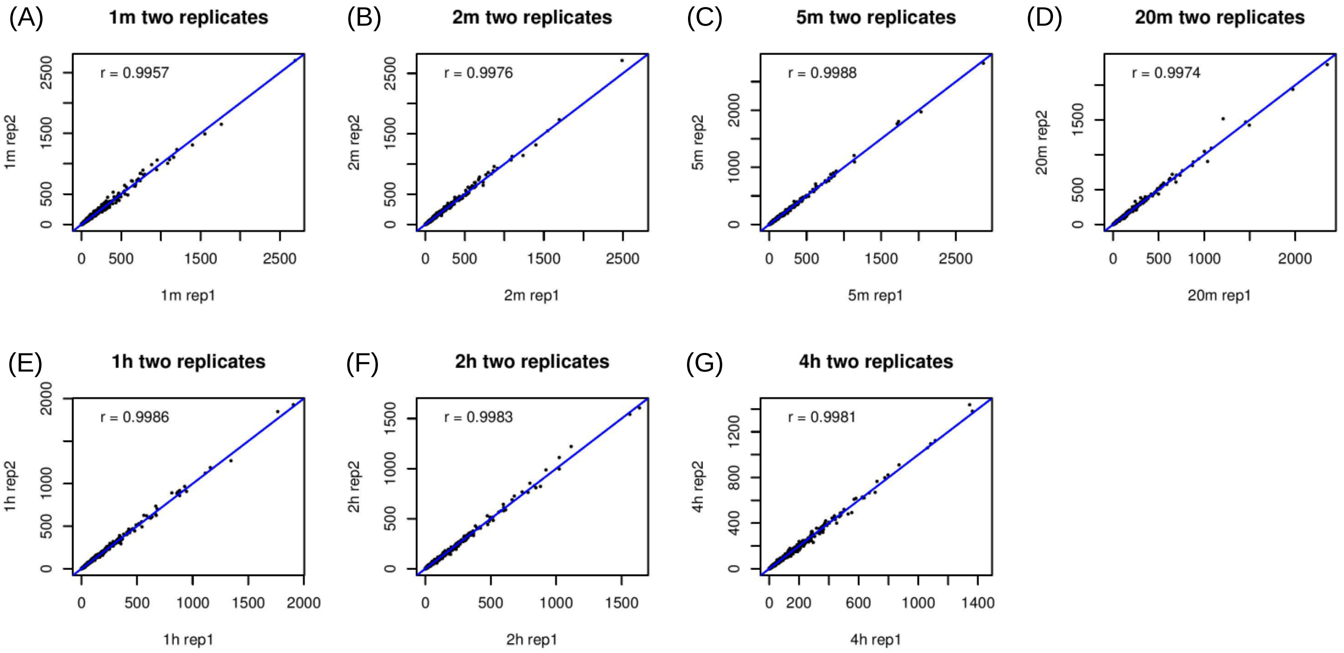

Figure S2: Read length distribution and di-nucleotide frequency of XR-seq data. Lengths of the excised oligomers fall within the range of 21 to 31 bp across all samples with peak at 26-27nt; TT/TC dinucleotides are enriched at the damage/repair sites by XR-seq. Three samples from early and late timepoints are shown: (A) (6-4)PP XR-seq 1 min replicate 1; (B) (6-4)PP XR-seq 4 h replicate 1; and (C) CPD XR-seq 12 min.

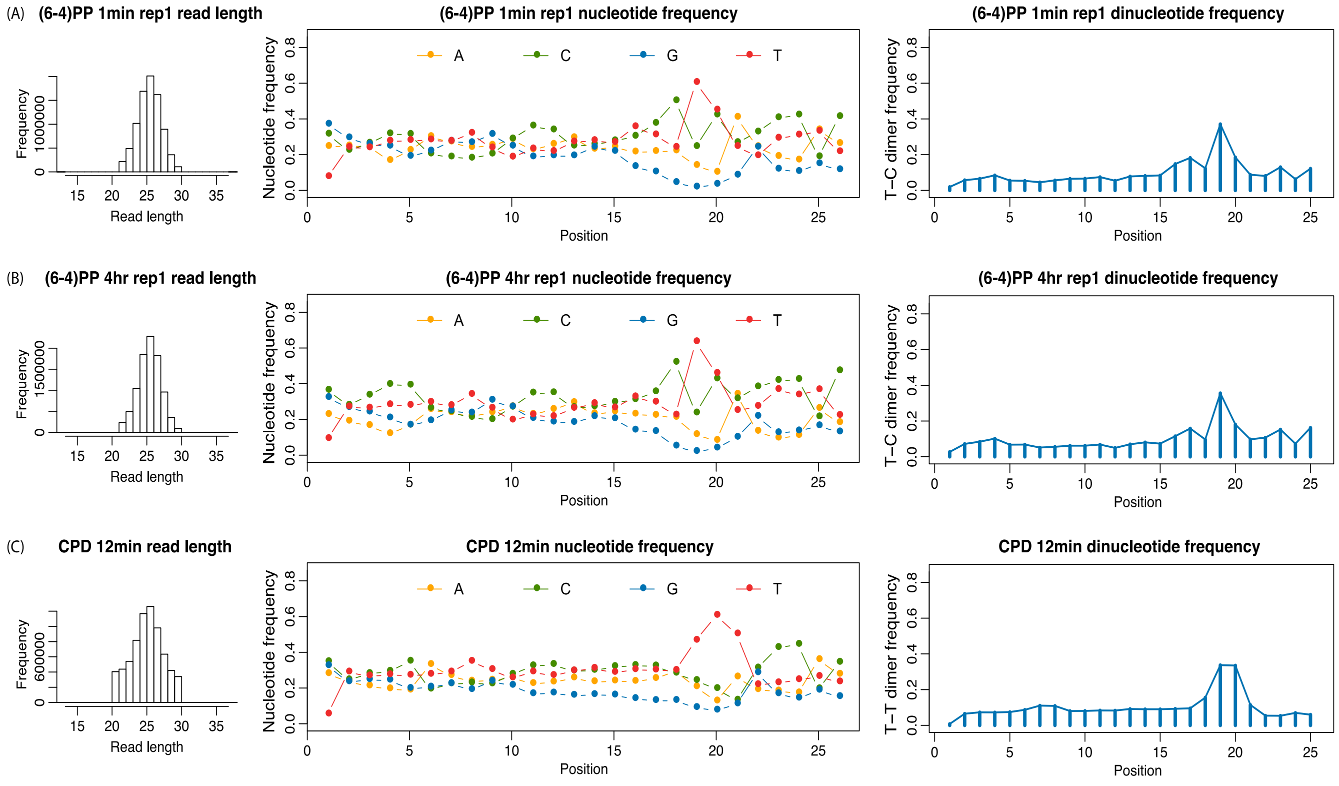

Figure S3: Excision repair in transcribed strand (TS) versus non-transcribed strand (NTS). For each gene, we quantify its strand-specific repair levels by XR-seq. Results are shown for (A) (6-4)PP XR-seq 1 min replicate 1, (B) (6-4)PP XR-seq 4hr replicate 1, and (C) CPD XR-seq 12 min. On the genome-wide scale, excision repair of damage on the TS and on the NTS is essentially equal, indicating no significant transcription-coupled repair of (6-4)PP at any timepoints and of CPD at the early 12 min timepoint.

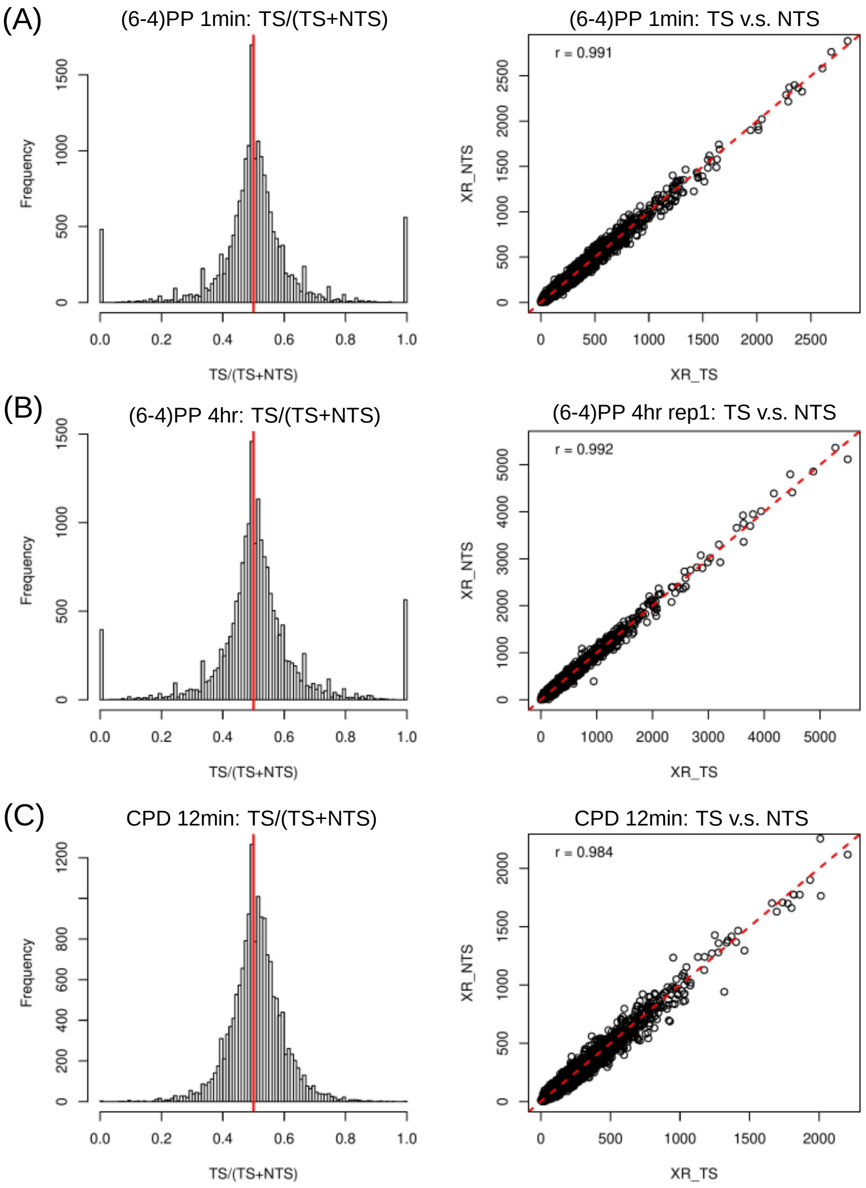

Figure S4: Downsampled depth of coverage for the identified super-hotspots and -coldspots across all timepoints. The depth of coverage is shown in a heatmap, where each row is a sample and each column is an identified hotspot or coldspot, with strand and chromosome annotations. (A) (6-4)PP repair hotspots. (B) (6-4)PP repair coldspots. (C) CPD repair hotspots. The depth of coverage is downsampled to remove biases due to different library size.

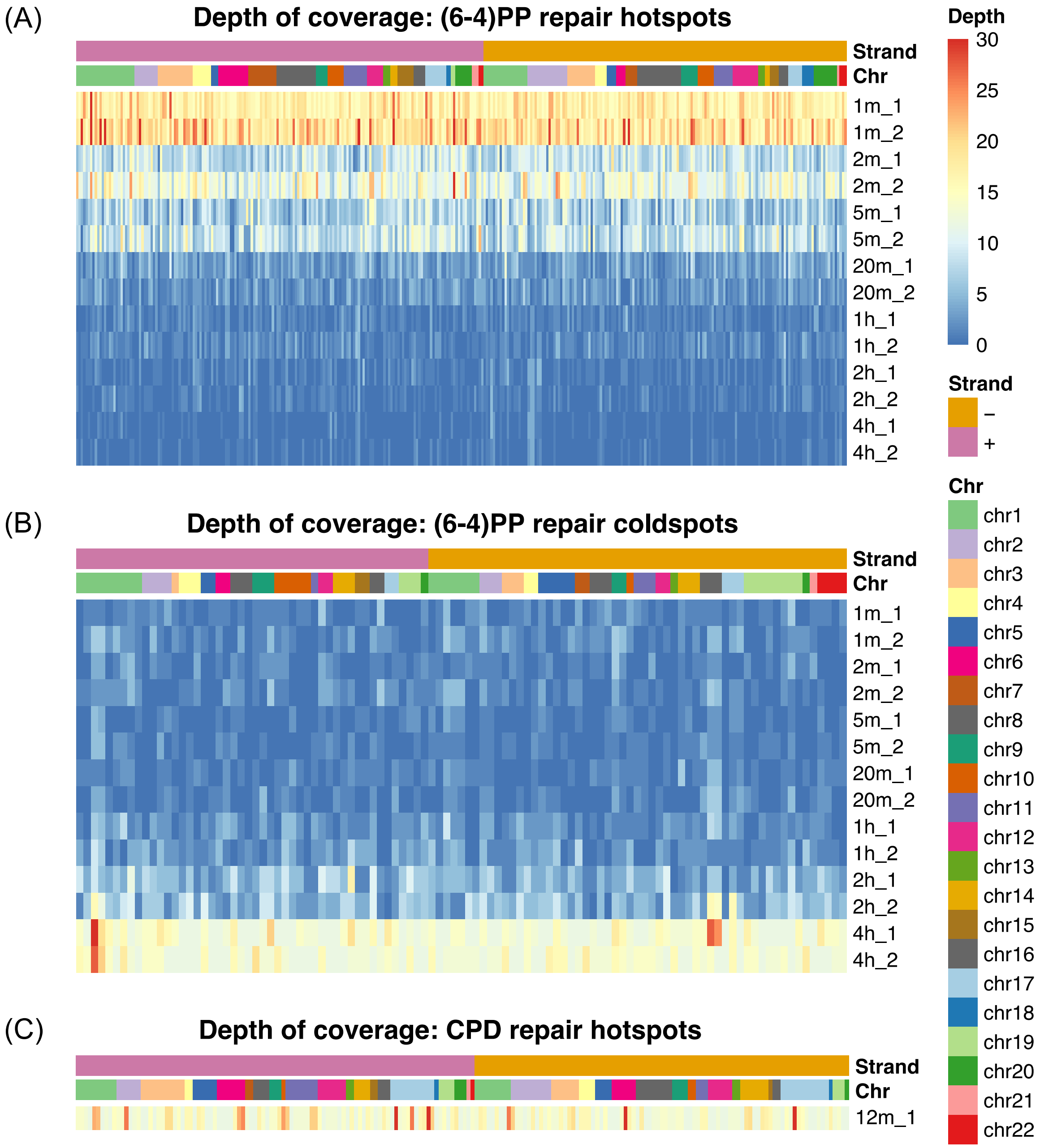

Figure S5: Normalized *z*-scores from Poisson log linear model for (6-4)PP repair. A cross-sample Poisson log linear model is applied to the repair data to account for library size difference, bin-specific artifacts, and TC content bias. The model is applied to the (A) plus and (B) minus strand separately for normalization. The detected repair super-hotspots and -coldspots harbor a much higher level of the observed repair (Y) than that of the null repair as expected (λ), in the 1 min and 4 hr replicates, respectively.

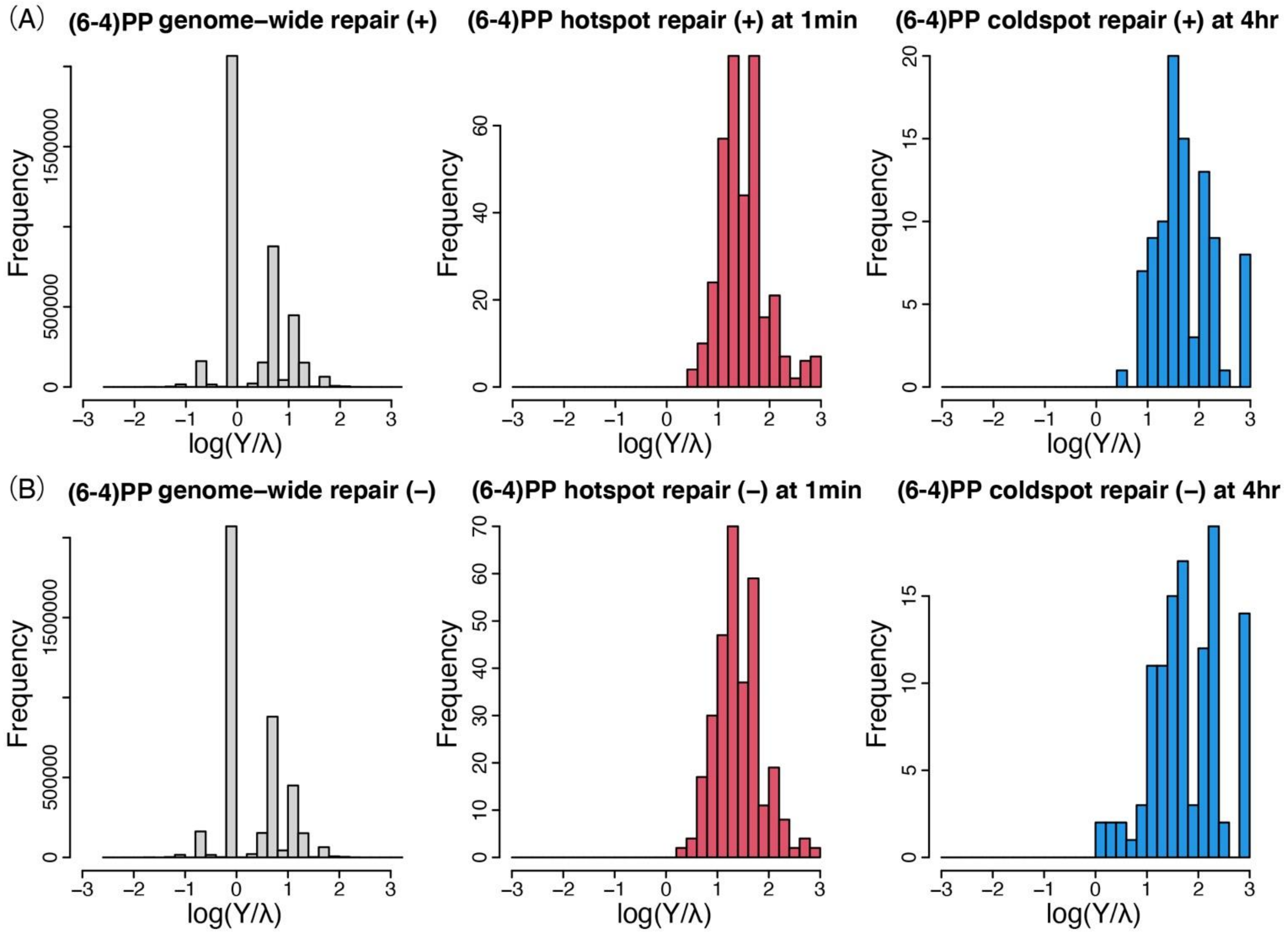

Figure S6: Genome-wide distribution of identified repair super-hotspots and -coldspots. Repair super-hotspots for (6-4)PP and CPD, and repair super-coldspots for (6-4)PP are separated by strands and visualized on chromosomal ideograms using Phenogram[1]. The super-hotspots and -coldspots are distributed across the entire genome fairly homogeneously.

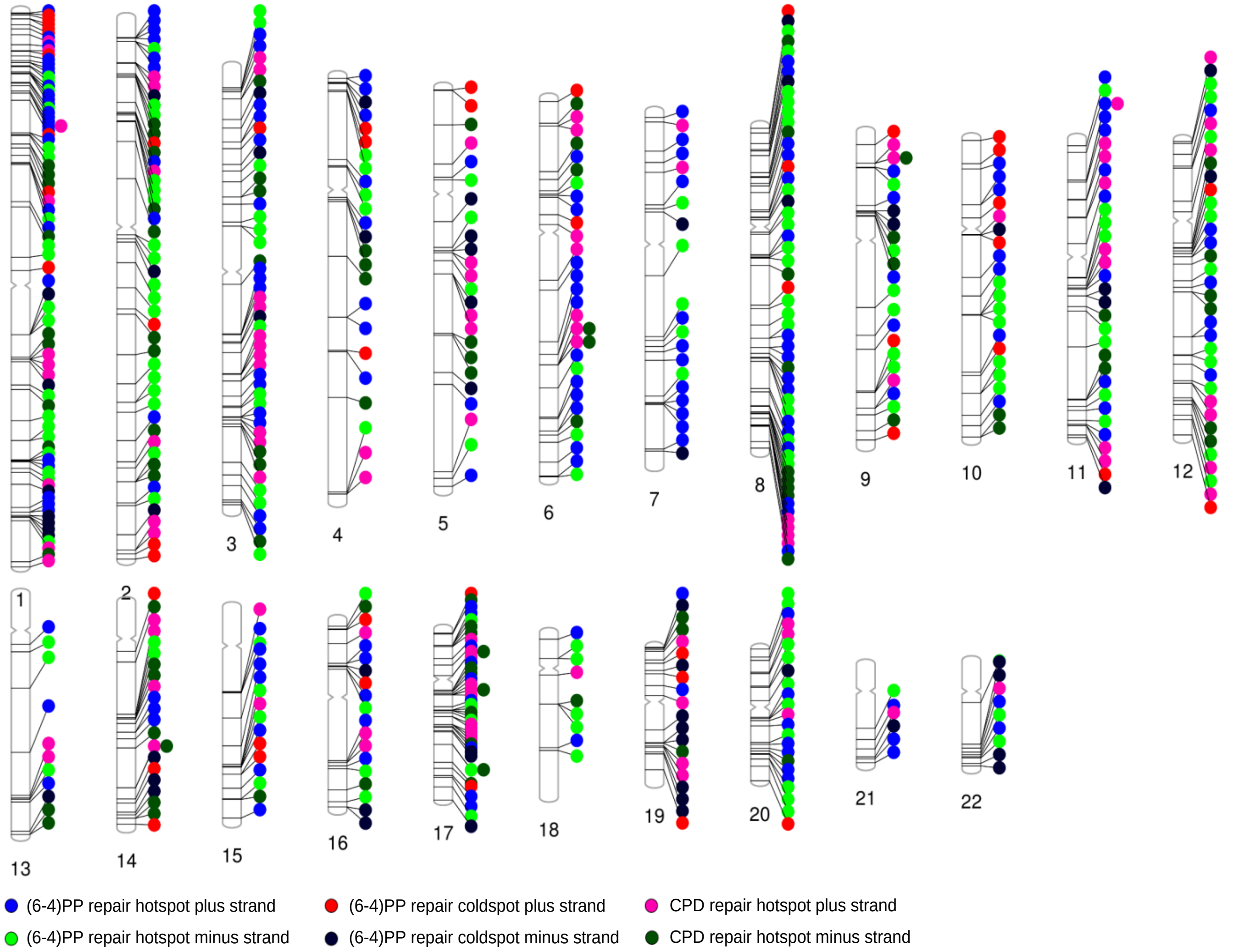

Figure S7: Epigenomic markers and genome-wide annotations for CPD damage hotspots. (A) DNase I hypersensitivity by DNase-seq and histone modifications by ChIP-seq bear no statistical differences between the CPD damage hotspots and random spots. (B) The identified damage hotspots are categorized based on chromatin states annotation, CpG-island-based annotation, and genic annotation, respectively. In each panel, the percentages of the damage hotspots from different annotated categories are shown as frequencies on the y-axis. CPD damage hotspots are enriched for heterochromatin regions, a finding that is concordant with previous reports[2, 3]. The damage hotspots are also enriched for inter-CpG and intronic regions, both of which are AT rich.

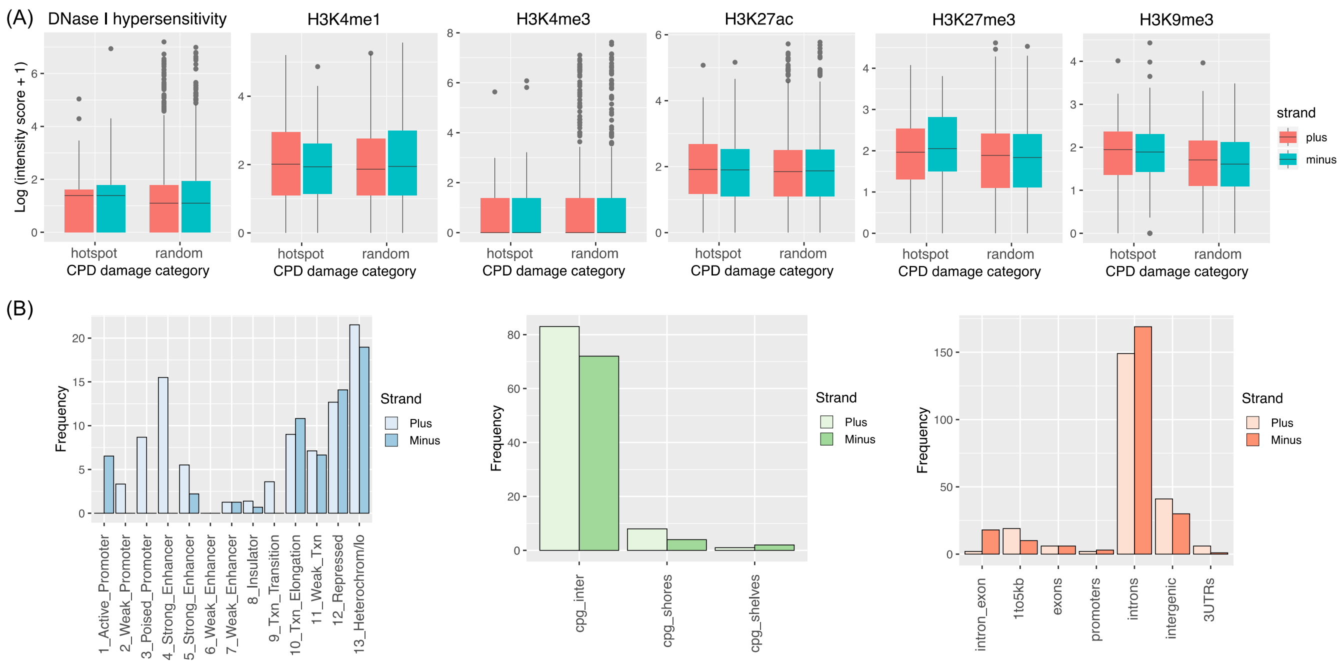

Figure S8. Genome-wide repair super-hotspots and -coldspots are associated with epigenomic markers. Profiles of chromatin accessibility (measured by DNase-seq as DNase I hypersensitivity) and histone modification (measured by ChIP-seq) for super-hotspots and -coldspots are shown for (A) (6-4)PP and (B) CPD repair. Chromatin accessibility is higher for repair super-hotspots and lower for super-coldspots. Repair super-hotspots are also characterized by higher ChIP-seq signals for H3K4me1, H3K4me3, and H3K27ac, markers for gene activations. Gene repression markers – H3K27me3 and H3K9me3 – do not tend to separate the different repair categories.

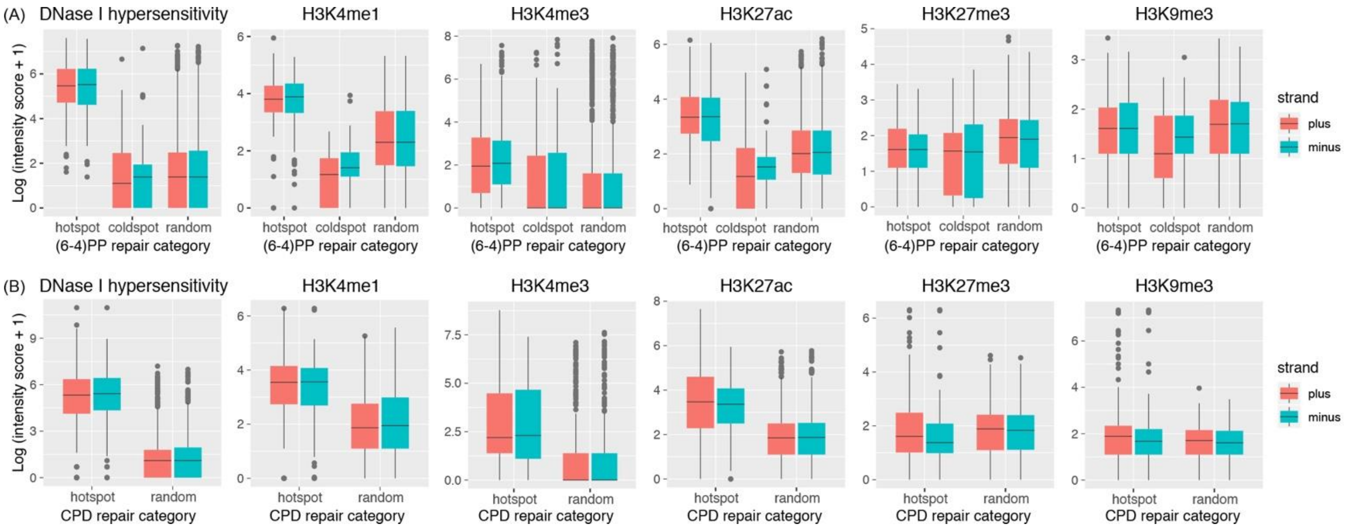

Figure S9: Genome-wide repair super-hotspots are enriched for enhancers. Annotations for (A) chromatin states, (B) CpG islands, and (C) genic/intergenic regions are shown for repair super-hotspots for (6-4)PP (top) and CPD (bottom), separated by strands. In each panel, the percentages of the damage hotspots from different annotated categories are shown as frequencies on the y-axis. Repair super-hotspots are enriched for enhancers and promoters, which are in open-chromatin regions. Repair super-hotspots are also enriched in inter-CpG and intronic regions, both of which are AT-rich.

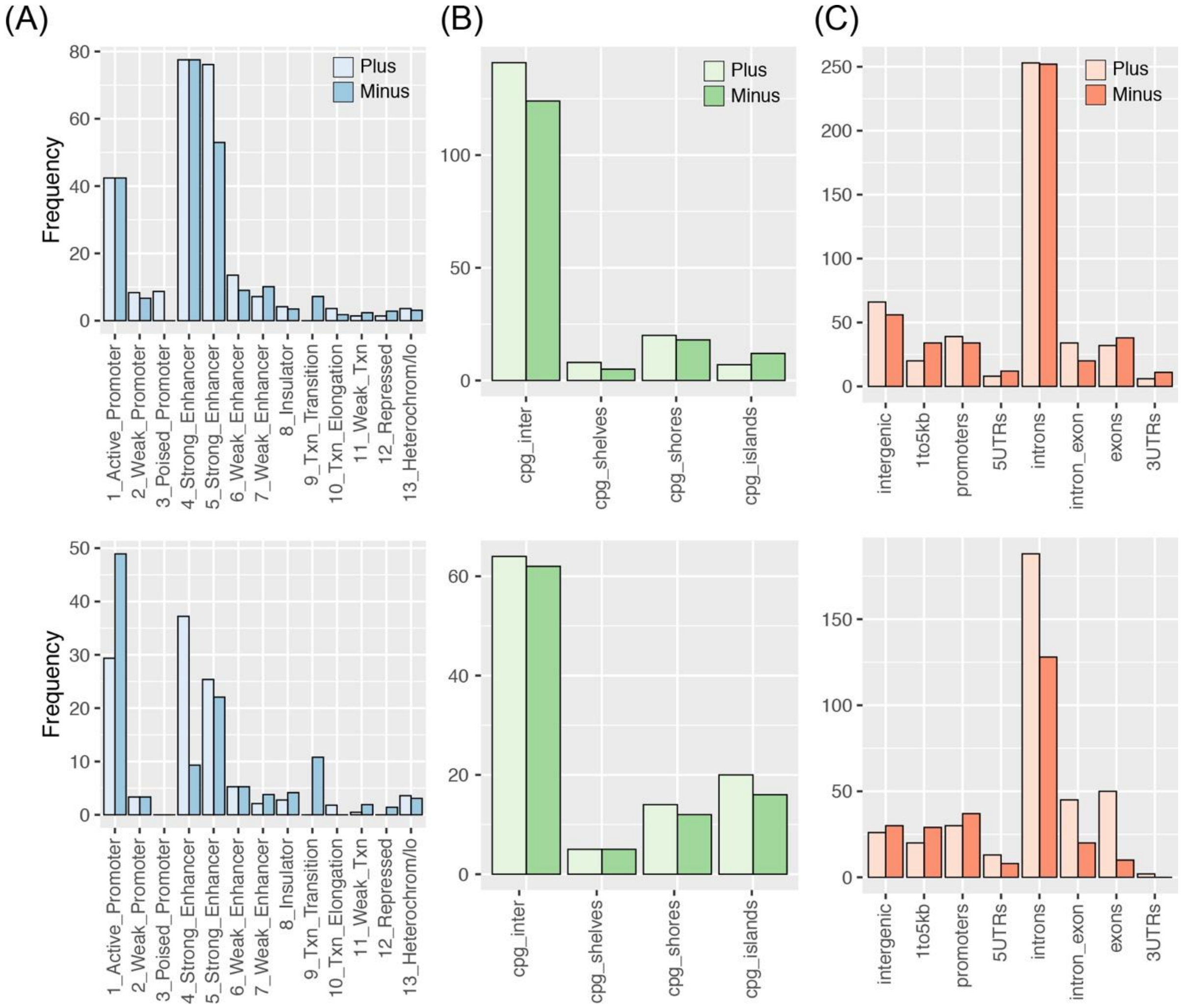

Figure S10: Genome-wide repair kinetics across timepoints for strong enhancers, weak enhancers, repressed regions, and heterochromatin regions. Enhancer regions are repaired at earlier timepoints, while repressed/heterochromatin regions are repaired at later timepoints. For strong enhancer and weak enhancer, the peaks at 5 min may be due to accumulation of non-degraded excision products from 1 min and 2 min.

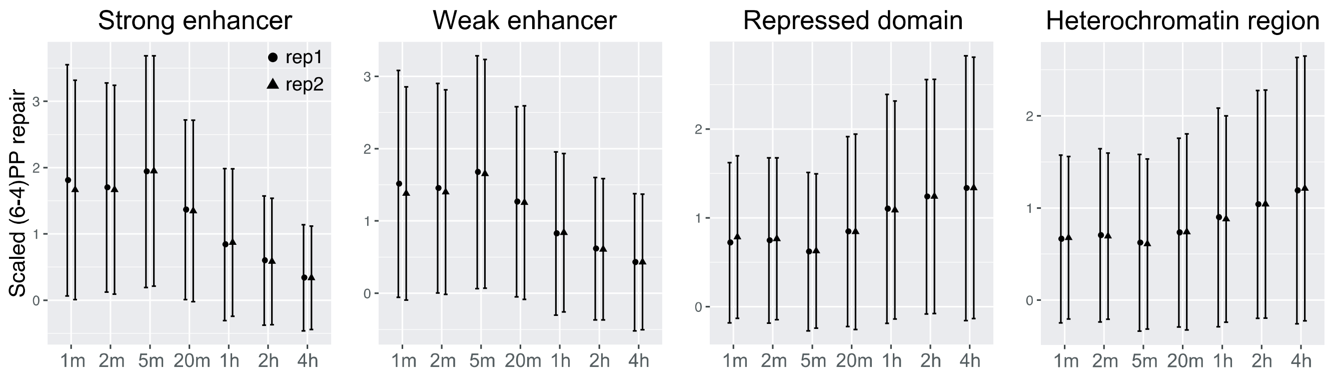

Figure S11: Genome-wide repair super-hotspots are enriched for early-replication domains. Replication timing domains, including early replication domains (ERDs), late replication domains (LRDs), down transition zones (DTZs), and up transition zones (UTZs), were identified using Repli-Seq data. The ‘replication-domain’ model states that ERDs and LRDs, connected by UTZs and DTZs, are spatially compartmentalized structural and functional units of higher-order chromosomal structure. Compared to genome-wide average (shown as the random category), there is a significantly higher proportion of the repair super-hotspots located in the early-replication domains.

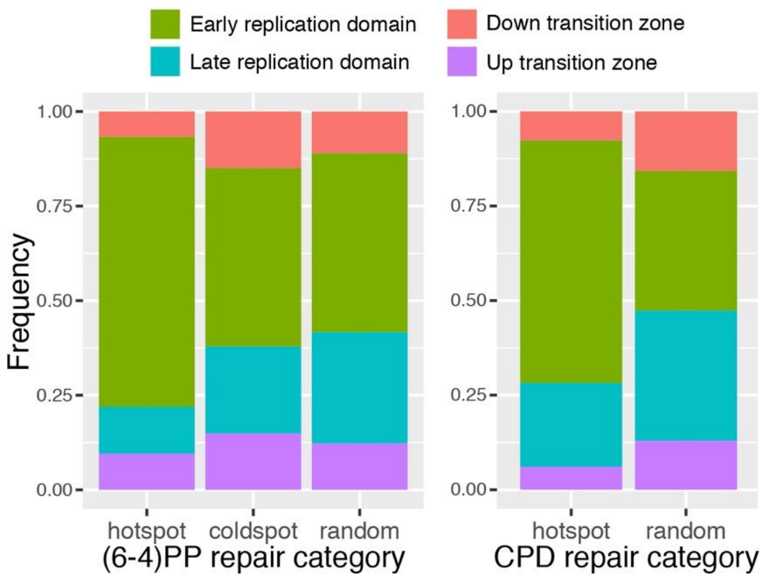

Figure S12: Repair super-hotspots overlap multiple consecutive enhancers from the same genomic regions. (A) An example minus strand repair super-hotspot from chr 2. (B) An example plus strand repair super-hotspot from chr 1. Both super-hotspots overlap super-enhancers.

(A)

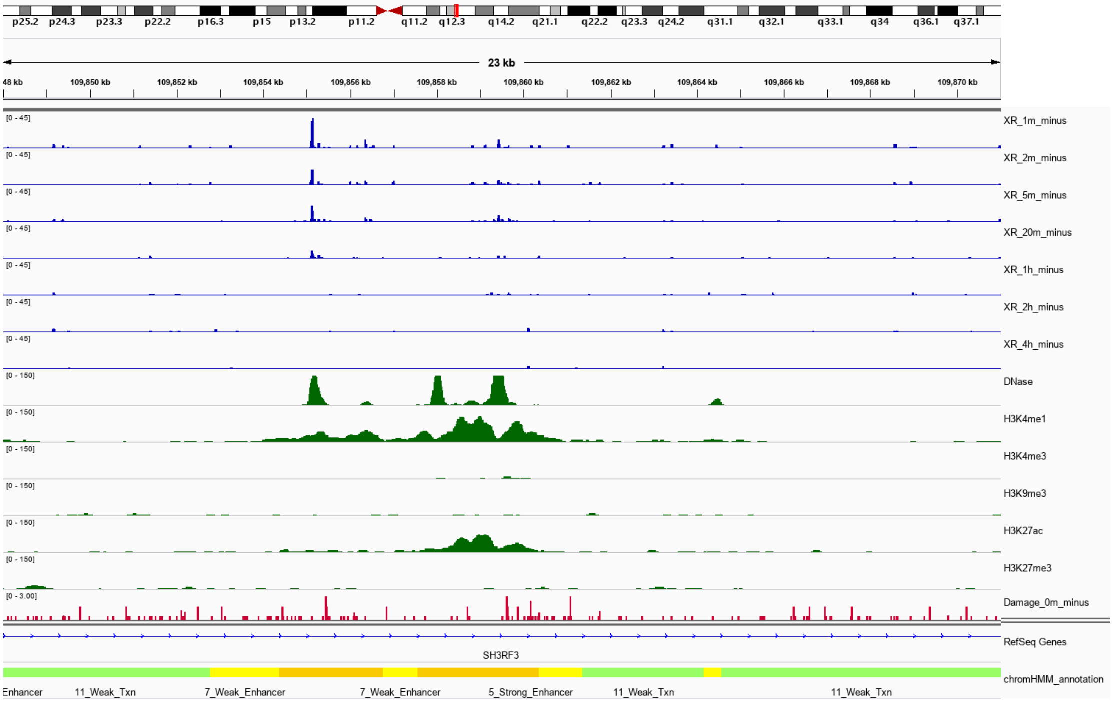

(B)

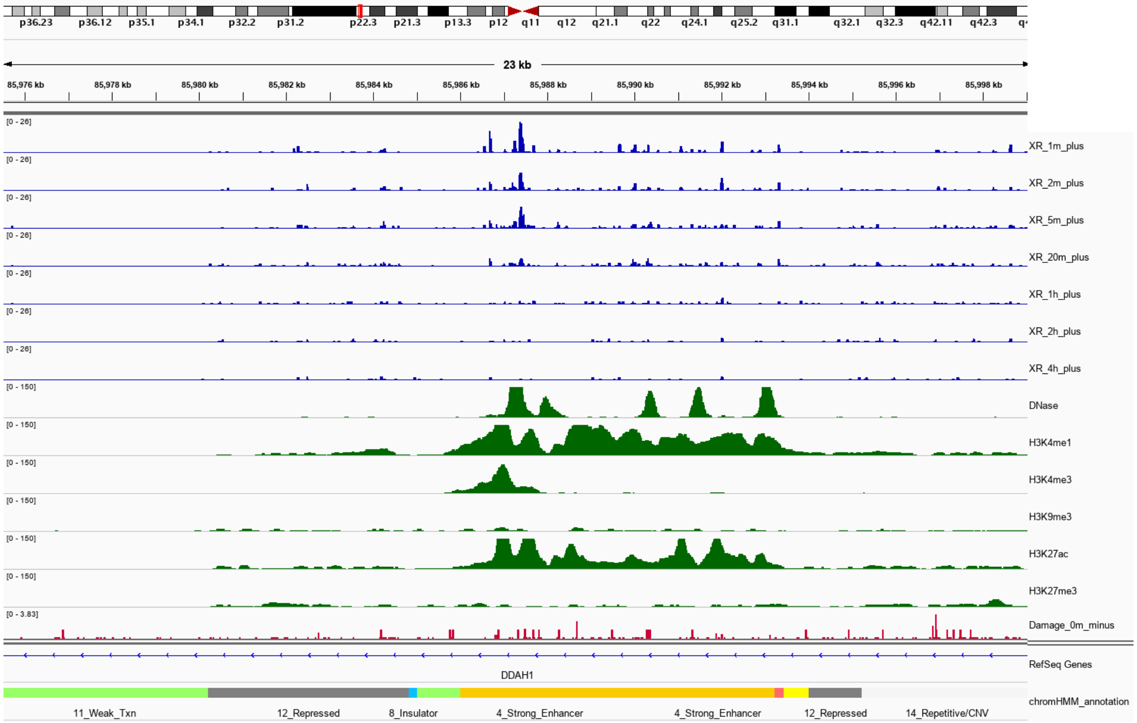

Figure S13: Nucleotide frequency of cytosine in repair super-hotspots and -coldspots compared with genomic average. Histograms of percentage of cytosine are shown (A) across all genomic bins, (B) in (6-4)PP repair super-hotspots, (C) in (6-4)PP repair super-coldspots, and (D) in CPD repair super-hotspots. The identified repair super-hotspots and -coldspots are enriched for cytosines in the flanking regions of the repair sites.

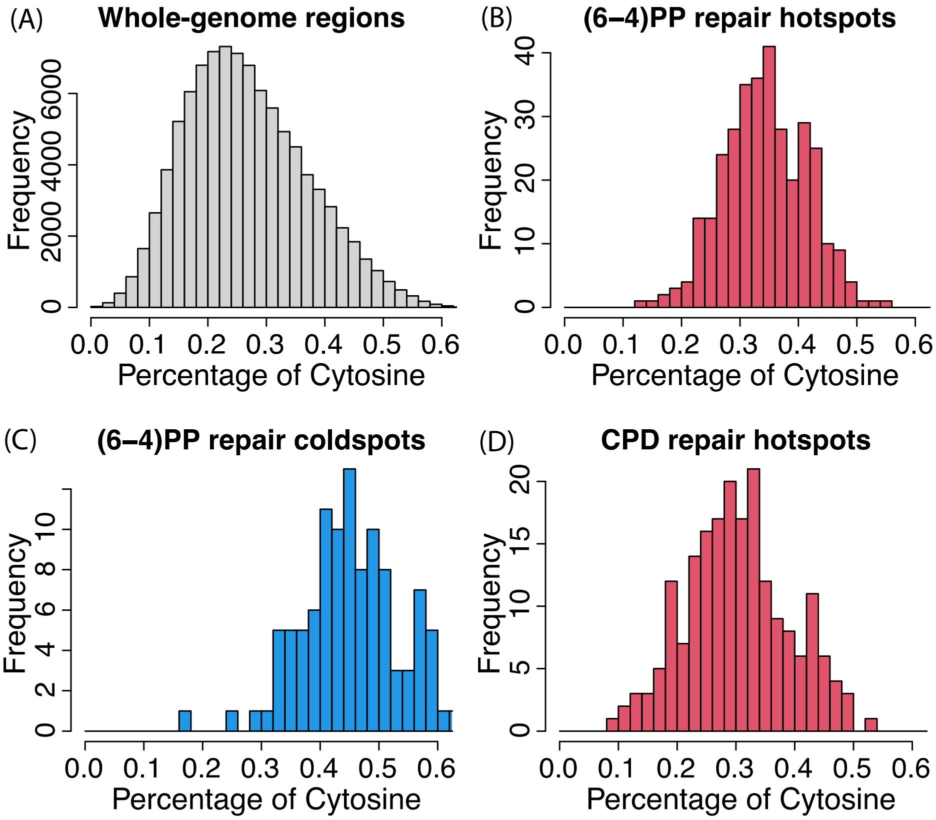

Table S1: XR-seq sample information. Number of reads is reported across all samples after each filtering step: fastq_reads is for the number of sequence reads in the raw sequencing file; total_mapped is for the total number of mapped reads; dedup is for the number of reads after deduplication; mapq is for the reads with mapping quality greater than 20; chr is for the reads that are mapped to the autosomes and the sex chromosomes; qwidth is the number of reads with lengths between 21 and 31bp; and genebody is the number of reads that are mapped to the gene bodies.

fastq_name	antibody	treatment	time	replicate	fastq_reads	total_mapped	dedup	mapq	chr	qwidth	genebody
**N641MR1_CAGATC_S49_L008_R1_001.fastq**	64	20J	1m	1	384802940	71402356	8401818	7941024	7930102	7930102	4326493
**N641MR2_GATCAG_S50_L008_R1_001.fastq**	64	20J	1m	2	422824212	67345807	8620110	8112862	8075112	8075112	4444832
**N642MR1_ACTTGA_S51_L008_R1_001.fastq**	64	20J	2m	1	279335280	53197655	23345765	22078323	22059768	22059768	11784233
**N642MR2_TAGCTT_S52_L008_R1_001.fastq**	64	20J	2m	2	248034148	45543788	16644732	15692341	15672060	15672060	8526503
**N645MR1_ATCACG_L005_R1_001.fastq**	64	20J	5m	1	104591936	19364359	16600527	15712745	15698665	15698665	8578429
**N645MR2_TGACCA_L005_R1_001.fastq**	64	20J	5m	2	82008780	15249676	12982483	12273842	12263361	12263361	6760420
**N6420MR1_ACAGTG_L003_R1_001.fastq**	64	20J	20m	1	146127236	26903762	20799106	19638269	19620576	19620576	10428629
**N6420MR2_GATCAG_L003_R1_001.fastq**	64	20J	20m	2	214152044	39984681	32266738	30521020	30494421	30494421	16161219
**N641HR1_TAGCTT_L003_R1_001.fastq**	64	20J	1h	1	194350240	34956862	30108510	28313194	28282892	28282892	14298448
**N641HR2_GCCAAT_L003_R1_001.fastq**	64	20J	1h	2	140819580	25008097	19975225	18762428	18741270	18741270	9587793
**N642HR1_TAGCTT_L001_R1_001.fastq**	64	20J	2h	1	60324552	10548055	9562799	8959193	8945613	8945613	4334201
**N642HR2_TTAGGC_L001_R1_001.fastq**	64	20J	2h	2	106209304	18167300	15697254	14698388	14679905	14679905	7102953
**N644HR1_GGCTAC_L001_R1_001.fastq**	64	20J	4h	1	60961524	10377267	9112994	8518484	8501855	8501855	3849252
**N644HR2_TGACCA_L001_R1_001.fastq**	64	20J	4h	2	99967420	16902160	13548187	12648710	12626997	12626997	5687242
**NHF1CPD12minrep1.fastq**	CPD	20J	12m	1	226271720	22029959	9193596	8146640	8146640	7002646	3659658

Table S2: (6-4)PP repair super-hotspots. Genomics coordinates and read depths for (A) 175 repair super-hotspots from the plus strand and (B) 156 repair super-hotspots from the minus strand. Separately attached as an excel file.

Table S3: (6-4)PP repair super-coldspots. Genomics coordinates and read depths for (A) 48 repair super-coldspots from the plus strand and (B) 57 repair super-coldspots from the minus strand. Separately attached as an excel file.

Table S4: CPD repair super-hotspots. Genomics coordinates and read depths for (A) 99 repair super-hotspots from the plus strand and (B) 93 repair super-hotspots from the minus strand. Separately attached as an excel file.

Table S5: CPD damage hotspots. Genomics coordinates and read depths for (A) 91 damage hotspots from the plus strand and (B) 78 damage hotspots from the minus strand. Separately attached as an excel file.

Table S6: Profiled FIRES, super-enhancers, and interactions using Hi-C data of human fibroblasts. (A) FIREs calling results by FIREcaller[4] using Hi-C data of IMR90[5]. Outputs include continuous FIRE scores, dichotomous FIREs, and super-FIREs. (B) List of super-enhancers of human fibroblasts, inferred by Hi-C data of IMR90[6]. (C) Significant interactions inferred from the Hi-C data[7]. Separately attached as an excel file.

Table S7: Repair super-hotspots and -coldspots overlapped FIREs, super-enhancers, and significant interactions. Number of overlapped FIREs, super-enhancers, and significant interactions for (A) (6-4)PP repair super-hotspots, (B) (6-4)PP repair super-coldspots, and (C) CPD repair super-hotspots. Separately attached as an excel file.

References

1. Wolfe D, Dudek S, Ritchie MD, Pendergrass SA. Visualizing genomic information across chromosomes with PhenoGram. *BioData Min* **6**, 18 (2013).

2. Han C, Srivastava AK, Cui T, Wang QE, Wani AA. Differential DNA lesion formation and repair in heterochromatin and euchromatin. *Carcinogenesis* **37**, 129-138 (2016).

3. Hauer MH, Gasser SM. Chromatin and nucleosome dynamics in DNA damage and repair. *Genes Dev* **31**, 2204-2221 (2017).

4. Crowley C*, et al.* FIREcaller: an R package for detecting frequently interacting regions from Hi-C data. *bioRxiv*, 619288 (2019).

5. Jin F*, et al.* A high-resolution map of the three-dimensional chromatin interactome in human cells. *Nature* **503**, 290-294 (2013).

6. Schmitt AD*, et al.* A Compendium of Chromatin Contact Maps Reveals Spatially Active Regions in the Human Genome. *Cell reports* **17**, 2042-2059 (2016).

7. Giusti-Rodríguez P*, et al.* Using three-dimensional regulatory chromatin interactions from adult and fetal cortex to interpret genetic results for psychiatric disorders and cognitive traits. *bioRxiv*, 406330 (2019).
